# The association between the triglyceride-glucose index and diabetic peripheral neuropathy: a systematic review and meta-analysis

**DOI:** 10.3389/fendo.2026.1901899

**Published:** 2026-09-04

**Authors:** Xin Gu, Maoying Wei, Chan Wu, Aijing Li, Anning Sun, Jingyi Guo, Haoshuo Wang, Churan Wang, Yincheng Li, Qingyi Zhu, Yanbing Gong

**Affiliations:** Dongzhimen Hospital, Beijing University of Chinese Medicine, Beijing, China

**Keywords:** diabetic peripheral neuropathy, insulin resistance, meta-analysis, systematic review, triglyceride glucose index

## Abstract

**Background:**

Diabetic peripheral neuropathy (DPN) is a prevalent and severe microvascular complication of type 2 diabetes mellitus (T2DM). Early risk screening and warning are critical for slowing disease progression. The triglyceride-glucose (TyG) index, a simple and efficient surrogate marker for assessing insulin resistance (IR), has been proven to be closely associated with various diabetic complications. However, existing studies on the association between the TyG index and the risk of DPN have yielded inconsistent findings, and relevant evidence still lacks systematic synthesis.

**Methods:**

A systematic review and meta-analysis were conducted. We systematically searched databases including PubMed, Embase, Web of Science, CNKI, Wanfang, and CQVIP to enroll observational studies exploring the association between the TyG index and the risk of DPN in patients with T2DM. Two investigators independently performed literature screening, data extraction, and quality assessment. The random-effects and fixed-effects models were applied to pool effect sizes, and subgroup analyses were carried out to explore potential sources of heterogeneity.

**Results:**

A total of 14 studies involving 32,281 participants were included. The results showed that a higher TyG index was associated with an increased risk of DPN, with a pooled odds ratio (OR) of 2.614 (95% CI: 1.788-3.821, *P* < 0.0001) and a pooled hazard ratio (HR) of 1.248 (95% CI: 1.003-1.553, *P* = 0.0471). This positive correlation remained consistent across most subgroups stratified by age, sample size, BMI, and adjustment for confounding factors. However, the association did not reach statistical significance in the non-China subgroup.

**Conclusion:**

Elevated TyG levels are associated with an increased risk of DPN in patients with T2DM. However, the causal relationship requires further validation. These findings may serve as a reference for clinical screening of DPN risk.

**Systematic Review Registration:**

https://www.crd.york.ac.uk/prospero, identifier CRD420261373476.

## 1 Introduction

Diabetic peripheral neuropathy (DPN) is a prevalent chronic complication of diabetes, affecting roughly 30% of patients with type 2 diabetes (T2DM) globally (1). With a prevalence of over 50% among patients with a diabetes duration of more than 10 years, DPN serves as a major cause of diabetic foot lesions, amputation, and chronic pain (2). DPN progressively impairs both sensory and motor functions, increasing the risks of muscle weakness, gait instability, and fall-related injuries (3). In addition, it contributes to sleep disturbances, anxiety, and depression, substantially diminishing patients’ quality of life and elevating all-cause and cardiovascular mortality (3, 4). As the disease advances, annual medical expenditures are 2–3 times higher in DPN patients than in patients without diabetic complications, substantially adding to the global healthcare economic burden and representing a critical unmet need in modern diabetes management (5). Nevertheless, over 50% of patients with DPN have an insidious onset, predominantly characterized by small-fiber damage at the early stage (2). Patients may only present with mild paresthesia or even remain completely asymptomatic, and irreversible structural lesions are often already present upon clinical diagnosis (2, 6). Therefore, early screening and risk stratification are critically important clinically to slow DPN progression, lower disability and medical burden, and optimize clinical outcomes in patients with diabetes.

Currently, the clinical diagnosis of DPN relies primarily on nerve conduction tests and electromyography (7). However, this approach only detects large-fiber nerve damage and has extremely low sensitivity for small-fiber injury, which readily leads to missed early diagnoses and delayed optimal intervention windows (8). Intraepidermal nerve fiber density (IENFD) testing is currently the gold standard for assessing small-fiber damage, with satisfactory diagnostic accuracy (9). Nevertheless, it is an invasive procedure with low patient acceptance; more critically, it requires specialized pathology laboratories and is therefore unavailable in most countries (10). Corneal confocal microscopy, as a novel non-invasive diagnostic technique, can detect early micro-neurological damage, but its clinical utility is restricted by high cost, complex operation, and scarce equipment availability (10). Neuropad and Sudoscan are rapid, non-invasive modalities capable of assessing small-fiber function; however, the diagnostic performance reported in existing studies is highly heterogeneous, and no standardized reference thresholds have been established to support their widespread clinical adoption (11, 12). In addition, although serum biomarkers such as neurofilament light chain have shown promising potential, their value in large-scale screening and patient stratification has yet to be robustly validated (13, 14). In summary, despite their respective advantages, none of these emerging techniques has yet accumulated sufficient evidence and practicality for routine clinical use. There is an urgent clinical demand for low-cost, convenient, sensitive and stable biomarkers for early DPN screening to identify DPN prior to overt clinical symptoms, monitor disease progression and reduce long-term foot complications (7).

From a pathophysiological perspective, accumulating evidence indicates metabolic disturbances act as core drivers of DPN onset and progression, with insulin resistance (IR) representing the key pathological basis for various metabolic abnormalities (15, 16). Disorders of glucose and lipid metabolism impair IR signaling pathways, ultimately leading to IR; in turn, IR promotes hepatic gluconeogenesis, impedes peripheral glucose uptake in skeletal muscle, and facilitates the excessive mobilization of free fatty acids, thereby further exacerbating glucolipid metabolic disturbances (17). Persistent hyperglycemia triggers the polyol pathway and inflammatory responses, facilitates the accumulation of advanced glycation end-products(AGEs), and induces mitochondrial dysfunction (18). Combined with lipotoxicity and oxidative stress, these processes injure the endothelium of neural microvessels, disrupt neuroimmune signaling, cause axonal damage and myelin loss, and impair nerve regeneration (18). Moreover, as the core pathological hallmark of T2DM, IR can induce metabolic dysfunction in neural tissue independently of glycemic status, accelerating peripheral nerve damage and functioning as a pivotal mediator in the pathogenesis and progression of DPN (19). Consequently, assessment of IR status may provide novel insights for early risk identification and adjunctive diagnosis of DPN.

A variety of approaches have been developed to assess IR. These can be broadly categorized into direct measurement techniques, such as the hyperinsulinemic-euglycemic clamp (HEC) and the minimal model analysis of the intravenous glucose tolerance test (IVGTT), and indirect surrogate indices, including the homeostasis model assessment of insulin resistance (HOMA-IR), the quantitative insulin sensitivity check index (QUICKI), and the Matsuda index (16). Among these, the triglyceride-glucose (TyG) index, a simple IR surrogate calculated from fasting triglyceride and glucose levels, has been validated to correlate significantly with insulin sensitivity measured by the HEC technique (20). Although HEC and IVGTT are considered the gold standards for IR assessment, their clinical utility and applicability in large-scale population studies are limited by their invasive nature, technical complexity, high cost, and time-consuming procedures (16). Meanwhile, commonly used surrogate indices such as HOMA-IR rely on fasting insulin measurements, which are susceptible to interference from exogenous insulin therapy; QUICKI and the Matsuda index require multiple blood samplings during an oral glucose tolerance test, adding to the procedural burden (16). In contrast, the TyG index offers several practical advantages: it does not require insulin measurement, is calculated from a single fasting blood sample using routine clinical laboratory parameters, and is characterized by low cost, ease of implementation, robust reproducibility, and high stability (21). These features render it particularly suitable for large-scale population screening and clinical application in primary care settings. In recent years, multiple observational studies have investigated the association between the TyG index and DPN. Most studies have suggested that an elevated TyG index is significantly associated with an increased risk of DPN and remains an independent risk factor after adjustment for multiple confounding factors (22). The area under the receiver operating characteristic curve (AUC) for this index peaked at 0.79, showing moderate to good discriminative ability (22). However, some studies have also failed to identify any correlation with the TyG index in specific populations such as patients with newly diagnosed diabetes, rendering the relevant results somewhat controversial to date (23). Furthermore, substantial between-study heterogeneity in sample size, ethnicity, disease duration, and diagnostic criteria, together with limited statistical power, precludes definitive conclusions regarding the magnitude of the association and its generalizability to specific populations.

To date, the association between the TyG index and the risk of DPN has not been systematically synthesized or quantitatively evaluated. Moreover, existing evidence remains inconsistent, and the diagnostic value of the TyG index has been insufficiently explored. Accordingly, we performed this systematic review and meta-analysis to comprehensively search and synthesize eligible observational studies, quantitatively assess the association between elevated TyG index and DPN risk, and determine the clinical value of the TyG index as an early screening and risk stratification tool. We anticipate that our findings will yield evidence to inform future strategies for DPN prevention and management, ultimately aiming to alleviate DPN-associated disability and disease burden.

## 2 Methods

This systematic review and meta-analysis aims to investigate the association between the TyG index and DPN. The study was reported in accordance with the Preferred Reporting Items for Systematic Reviews and Meta-Analyses (PRISMA) 2020 statement **Supplementary Table 1** (24). The study protocol was registered with PROSPERO (registration ID: CRD420261373476).

### 2.1 Search strategy

We performed a comprehensive literature search across six databases: PubMed, EMBASE, Web of Science, CNKI, Wanfang, and CQVIP, to identify eligible observational studies investigating the association between the TyG index and DPN. The search strategy combined the following keywords and MeSH terms: “triglyceride-glucose index,” “TyG index,” “triacylglycerol glucose index,” “TyG,” “Diabetic Neuropathies,” “Diabetes Complications,” “Diabetic Neuralgia,” and “diabetic nerve damage.” The search was limited to studies published in English or Chinese between January 1, 2008, and July 29, 2026. The full search strategy is provided in **Supplementary Table 2**.

### 2.2 Inclusion criteria

1. Study design: Observational studies, including cohort, case-control, and cross-sectional studies.
2. Study population: Patients diagnosed with T2DM who were aged ≥ 18 years at baseline.
3. Exposure factor: Participants were grouped according to different TyG index levels at baseline. The TyG index was calculated as ln[(fasting triglyceride (mg/dL) × fasting plasma glucose (mg/dL)/2)] (25).
4. Outcome: DPN was defined as the outcome, and eligible studies were required to report effect estimates or provide sufficient data for their derivation. As no universal diagnostic criteria for DPN have been established, we adopted those applied in the original studies. All diagnostic criteria and standardized assessment scales complied with internationally recognized clinical guidelines (26–31).

### 2.3 Exclusion criteria

1. Duplicate publications, or studies with unavailable full texts;
2. Case reports, letters, narrative reviews, basic science studies, and conference abstracts;
3. Non-peer-reviewed preprints and dissertations;
4. Studies with insufficient data for effect estimation.

### 2.4 Literature screening and data extraction

Retrieved citations were imported into EndNote X9.1 (Clarivate Analytics, Philadelphia, PA, USA) for automatic deduplication. Two independent reviewers (A.L. and A.S.) independently screened the remaining citations in two phases: initial title and abstract screening, followed by full-text assessment based on predefined eligibility criteria. Any disagreements were resolved through consensus or by a third reviewer (J.G.) if consensus could not be reached. All final results were cross-checked by two independent investigators to ensure accuracy. Data extraction: The following information was extracted from each included study: (1) Study characteristics: first author, publication year, study design, sample size and data source; (2) Participant characteristics: age, sex, country and diabetes duration; (3) Outcome measures: DPN diagnostic criteria, effect estimates (odds ratios [ORs], hazard ratios [HRs]), descriptive data of the TyG index (mean, standard deviation [SD], median, interquartile range [IQR]), and diagnostic performance metrics (sensitivity, specificity, AUC, cut-off value). When multiple-adjusted models were reported in the included studies, the model with the most comprehensive adjustment for potential confounders was selected. The TyG index was analyzed as either a continuous or categorical variable in this meta-analysis. For studies reporting the TyG index categorically, the effect estimate comparing the highest quartile with the lowest quartile (reference group) was extracted. For studies reporting it continuously, the effect estimate per 1-unit increase in the TyG index was extracted.

### 2.5 Quality assessment

Two independent reviewers (C.W. and M.W.) assessed the risk of bias and methodological quality of the included studies. Any disagreements were resolved through discussion with a third investigator (H.W.). The Agency for Healthcare Research and Quality (AHRQ) tool was used to evaluate cross-sectional studies, with scores of 0-3, 4-7, and 8–11 indicating low, moderate, and high quality, respectively (32). The Newcastle-Ottawa Scale (NOS) was applied to cohort and case-control studies, with scores of 0-3, 4-6, and 7–9 representing low, moderate, and high quality, respectively (33).

### 2.6 Statistical analysis

All statistical analyses were conducted using R software (version 4.3.2) and Stata (version 16.0). Effect estimates were expressed as HRs or ORs with 95% confidence intervals (CIs) to quantify the association between the TyG index and DPN. Given that the TyG index was predominantly reported as a continuous variable with uniform measurement units across included studies, the pooled mean difference (MD) was employed to compare TyG index levels between the DPN and non-DPN groups. To explore the discriminative ability of the TyG index for DPN, we conducted an exploratory diagnostic analysis in addition to the primary association analyses. Given the small number of eligible diagnostic studies, we did not pool sensitivity and specificity; instead, we qualitatively summarized the diagnostic parameters from each included study. Meanwhile, a hierarchical summary receiver operating characteristic (HSROC) model was fitted to construct the HSROC curve and calculate the pooled AUC, visualizing the overall discriminatory performance of the TyG index for DPN. Spearman’s rank correlation test on logit-transformed sensitivity and 1-specificity was performed to assess the threshold effect. For studies lacking complete 2 × 2 contingency tables, relevant data were calculated from the reported sensitivity, specificity, and counts of patients and controls (34). For studies reporting only the median and IQR, we used the methods proposed by Wan et al. (35) and Luo et al. (36) to estimate the mean and SD. Heterogeneity was evaluated using Cochrane’s Q test and the *I²* statistic. A fixed-effects model with the inverse variance method was used when *P* ≥ 0.10 and *I²* < 50% (indicating low heterogeneity), whereas a random-effects model with the restricted maximum likelihood (REML) estimator was applied when *P* < 0.10 and *I²* ≥ 50% (indicating substantial heterogeneity). Prespecified subgroup analyses were conducted to explore potential sources of heterogeneity. Sensitivity analyses were performed using the leave-one-out method. Publication bias was assessed through visual inspection of funnel plots and Egger’s test. A two-sided *P* value < 0.05 was considered statistically significant.

## 3 Results

### 3.1 Literature screening process and results

**Figure 1:**
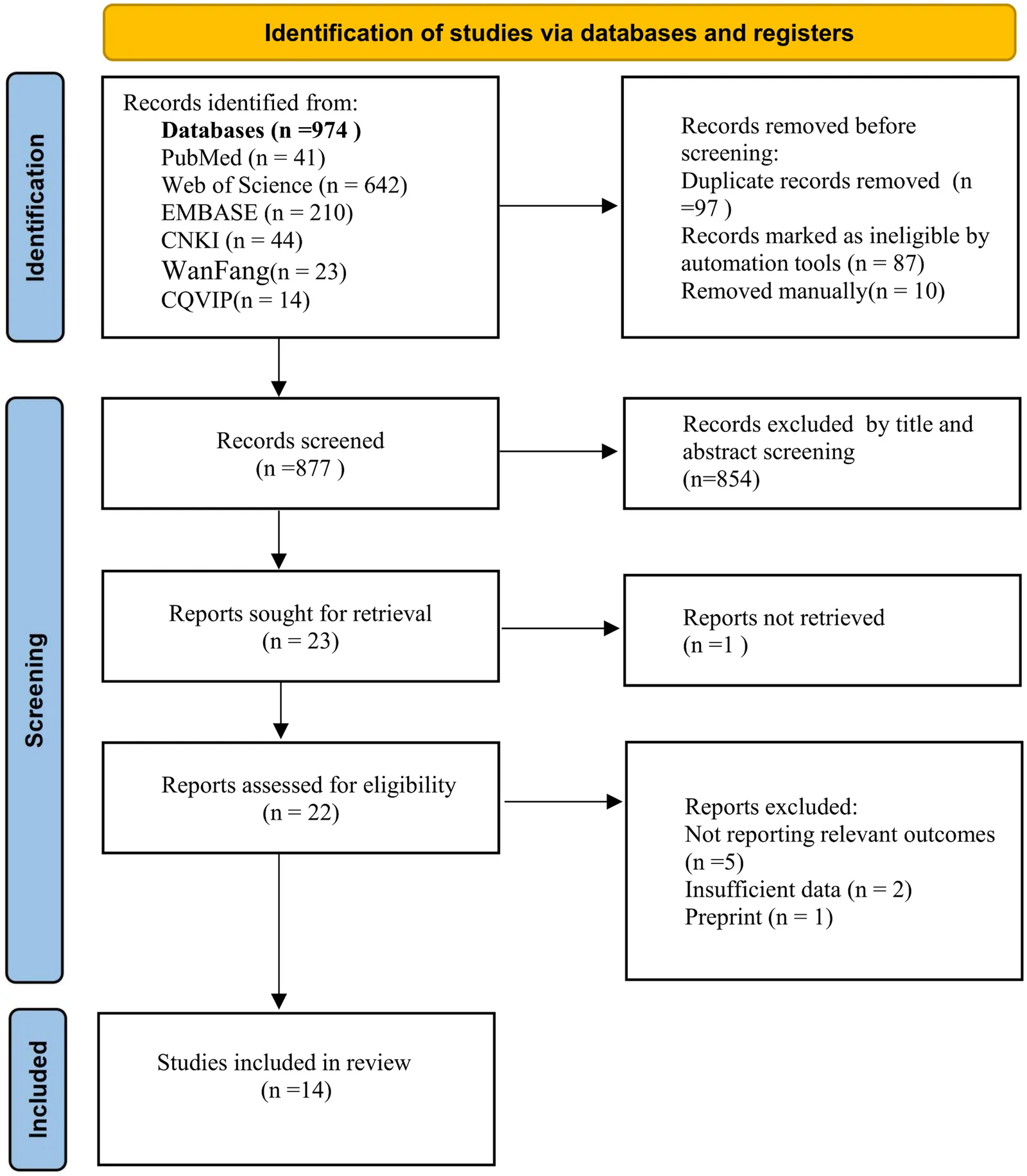
Flow chart of the study selection process.

A total of 974 records were identified through the preliminary search: 41 from PubMed, 642 from Web of Science, 210 from Embase, 44 from CNKI, 23 from Wanfang, and 14 from CQVIP. After title and abstract screening and full-text review, 14 studies (22, 23, 37–48) were ultimately included. The PRISMA flow diagram illustrating the selection process is shown in **Figure 1**.

### 3.2 Basic characteristics and quality assessment results of the included studies

Fourteen studies involving 32,281 participants were included: seven articles (23, 43–48) published in Chinese and seven studies (22, 37–42) in English. Among them, there were ten cross-sectional studies (22, 23, 41–48) and four cohort studies (37–40). These studies were carried out across eight countries: China, India, the United States, the United Kingdom, Canada, Egypt, Brazil, and Italy. The follow-up duration of cohort studies varied from 3.69 to 16.80 years, and diabetes duration ranged from 1.00 to 15 years. Eleven studies reported ORs (22, 23, 40–48), and three studies reported HRs (37–39). Quality assessment showed 4 studies (43–46) of moderate quality (AHRQ scores of 6-7) and ten studies of high quality (22, 23, 37–42, 47, 48) (six with AHRQ scores of 8-9, and four with NOS scores of 8-9). Baseline characteristics and detailed quality assessment results are presented in **Table 1** and **Supplementary Table 3**.

**Table 1:** Basic characteristics of included studies.

Study	Country	Study design	Male	Age	Sample size	TyG analysis type	Follow-up duration (years)	Data source	T2D duration(year)	AHRQ/ NOS
Cardoso CRL 2026 (37)	Brazilian	Cohort study	258	60.2 ± 9.5	667	Continuous	median follow-up of 10.6 years	Clementino Fraga Filho University Hospital	8 (3,15)	8
Cao BF 2025 (38)	U.S. andCanada	Cohort study	3057	62.4 ± 6.6	5000	Continuous	median follow-up of 3.69 years	BioLINCC	NR	9
Yuan XL 2026 (39)	United Kingdom	Cohort study	11876	61.00 (55,65)	19512	Continuous	median follow-up of 12.9 years	UK Biobank repository	5	8
Sbriscia M 2025 (40)	Italian Republic	Cohort study	NR	40-87	465	Continuous	median follow-up of 16.8 years	IRCCS INRCA hospital	NR	9
Kassab HS 2023 (22)	Egypt	Cross- sectional study	225	54.0 ± 8.6	500	Continuous	_	Alexandria Main University Hospital	9.1 ± 6.8	8
Miranda CS 2025 (41)	India	Cross- sectional study	61.23	61.23 ± 11.85	125	Continuous	_	Father Muller Medical College Hospital	7.82 ± 5.10	8
Tu ZH 2024 (42)	China	Cross- sectional study	1185	60 (48,66)	1931	Categorical	_	Tongren Hospital, Shanghai Jiao Tong University School of Medicine	5.25 (0.25,12.41)	9
CAI JY 2024 (23)	China	Cross- sectional study	221	47.48 ± 12.99	300	Continuous	_	Wenzhou People’s Hospital	1	8
Yang D 2025 (43)	China	Cross- sectional study	152	54.37 ± 11.82	220	Continuous	_	Anhui Provincial Hospital of Traditional Chinese Medicine	NR	6
Zhang D 2025 (44)	China	Cross- sectional study	224	59.86 ± 10.71	357	Continuous	_	The Third Affiliated Hospital of Jinzhou Medical University	11.97 ± 6.93	7
Mai HH 2025 (45)	China	Cross- sectional study	32	64.71 ± 8.83	68	Continuous	_	Luoyang Third People’s Hospital	NR	7
Xu Fang 2022 (46)	China	Cross- sectional study	145	56 (50,68)	239	Continuous	_	Taizhou People’s Hospital	NR	7
Deng B 2023 (47)	China	Cross- sectional study	1798	56.97 ± 10.76	2790	Categorical	_	The First Hospital of Nanchang	8.51 ± 0.96	8
Yang NN 2026 (48)	China	Cross- sectional study	64	66.23 ± 4.47	107	Continuous	_	Lu’an Hospital of Anhui Medical University	8.76 ± 2.82	8

AHRQ, Agency for Healthcare Research and Quality; BioLINCC, Biologic Specimen and Data Repository Information Coordinating Center; INRCA, Istituto Nazionale di Ricovero e Cura per Anziani; IRCCS, Istituto di Ricovero e Cura a Carattere Scientifico; NOS, Newcastle-Ottawa Scale; NR, Not Reported; T2DM, Type 2 Diabetes Mellitus; TyG, triglyceride glucose index.

### 3.3 The mean difference of TyG index in DPN vs. non-DPN groups

**Figure 2:**
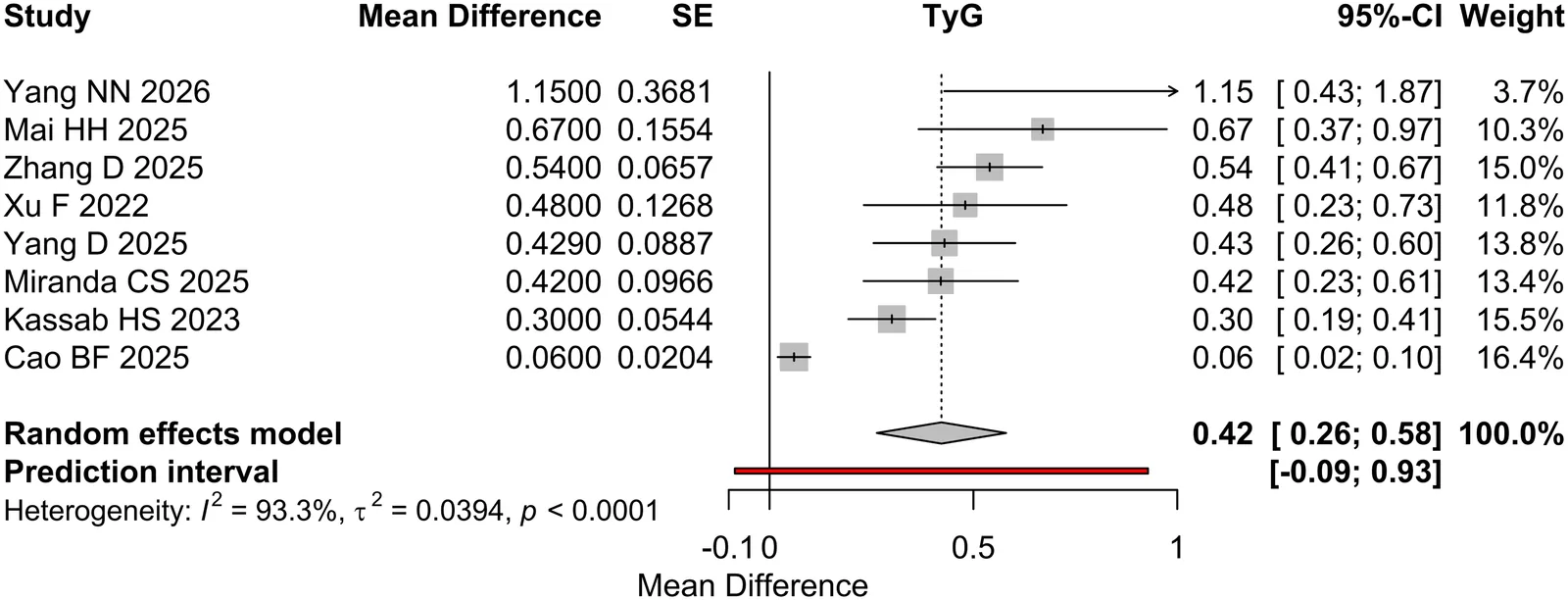
The mean difference of triglyceride-glucose index in DPN vs. non-DPN groups.

Eight studies (22, 38, 41, 43–46, 48) reported the mean and SD of the TyG index. Substantial heterogeneity was observed across these studies (*I²* = 93.3%, Q = 104.88, df = 7, *P* < 0.0001); therefore, a random-effects model was used to pool the MD. The pooled MD was 0.42 (95% CI: 0.26-0.58, Z = 5.21, *P* < 0.0001) (**Figure 2**), indicating that the TyG index was significantly higher in patients with DPN than in those without DPN. To explore potential sources of heterogeneity, we performed subgroup analyses stratified by age (< 60 or ≥ 60 years), region (Asian or non-Asian), sample size (< 500 or ≥ 500 participants), adjustment for confounding factors (Yes or No), and BMI (< 25 or ≥ 25 kg/m²). The pooled effect estimates were not statistically significant in the non-Asian region and the sample size ≥500 subgroup (*P* > 0.05) but were significant in the remaining subgroups (*P* < 0.05). Furthermore, significant between-subgroup differences in effect estimates were detected for region and sample size stratification (*P* = 0.0095), suggesting that geographic distribution and sample size may be potential sources of heterogeneity (**Supplementary Table 4**, **Supplementary Figure 1**).

### 3.4 Risk analysis of TyG index and DPN

**Figure 3:**
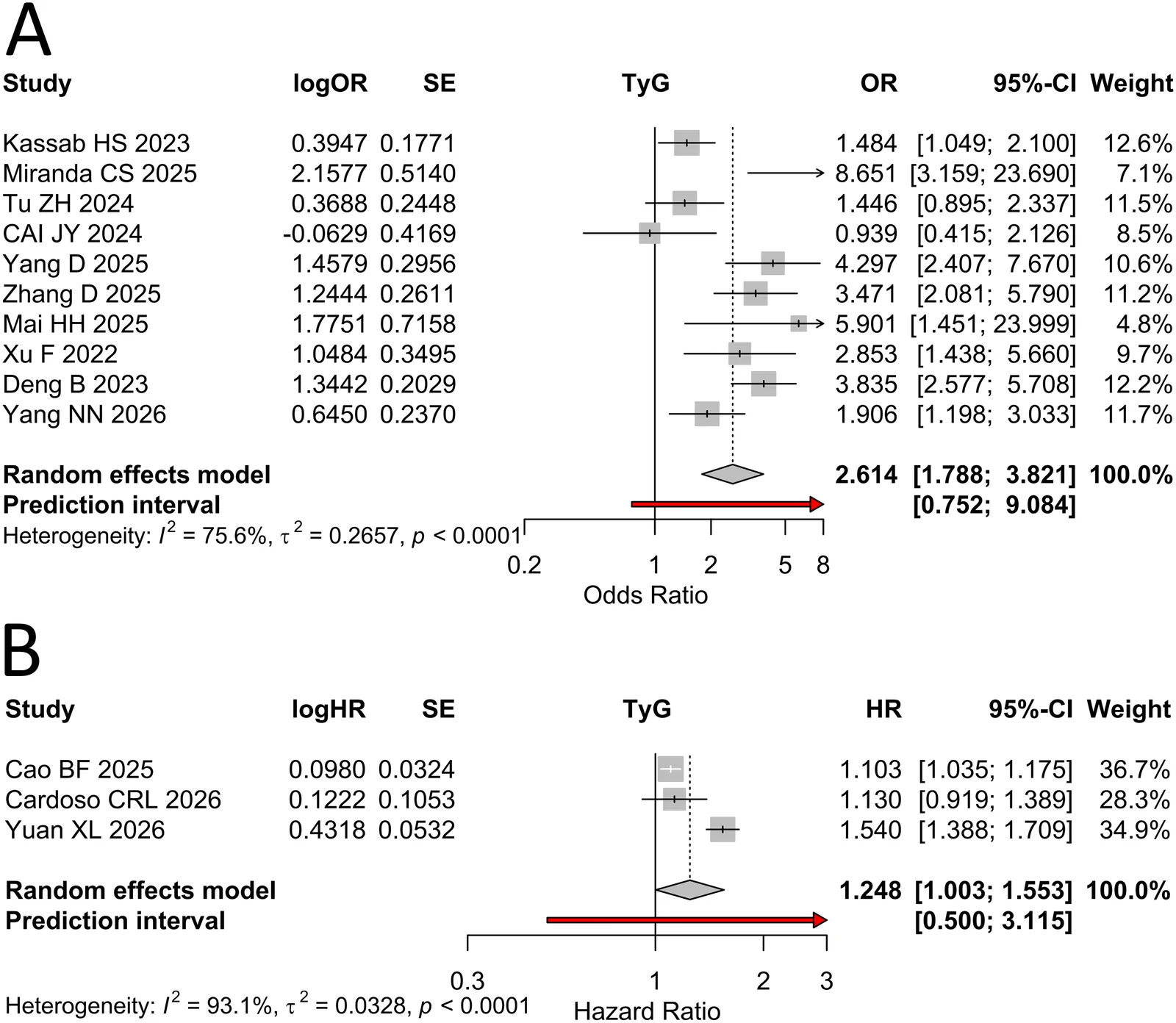
Forest plot for the association between triglyceride glucose index and risk of diabetic peripheral neuropathy. **(A)** odds ratio (OR) and **(B)** hazard ratio (HR).

Eleven studies (22, 23, 40–48) reported the association between the TyG index and DPN using OR, including 10 cross-sectional studies and 1 cohort study. Given the distinct risks of temporal bias between the two study designs and the insufficient sample size of the cohort study, quantitative meta-analysis pooling was only performed on cross-sectional studies in the present work. The OR of the single cohort study was 1.57 (95% CI: 1.03-2.41), which was excluded from the pooled analysis. Substantial heterogeneity was observed across these studies (*I^2^* = 75.6%, τ^2^ = 0.2657, *P* < 0.0001); therefore, a random-effects model was used to pool the effect estimates. The pooled analysis indicated that an elevated TyG index was significantly associated with an increased risk of DPN (OR = 2.614, 95% CI: 1.788-3.821, Z = 4.96, *P* < 0.0001) (**Figure 3A**). Subgroup analyses were conducted with respect to age (< 60 or ≥ 60 years), BMI (< 25 or ≥ 25 kg/m²), sample size (< 500 or ≥ 500 participants), study region (China or non-China), and adjustment for confounding factors (Yes or No) to identify potential sources of heterogeneity. The association remained significant in most subgroups, except the non-China subgroup, in which no statistically significant correlation was observed (OR = 3.354, 95% CI: 0.599-18.783, *P* = 0.1686) (**Supplementary Figure 2**; **Supplementary Table 5**).

Three studies (37–39) used HR to examine the association between the TyG index and incident DPN risk. The pooled analysis revealed a positive association between an elevated TyG index and DPN risk (HR = 1.248, 95% CI: 1.003–1.553, Z = 1.99, *P* = 0.0471) (**Figure 3B**).

### 3.5 Diagnostic performance of the TyG index for DPN

**Figure 4:**
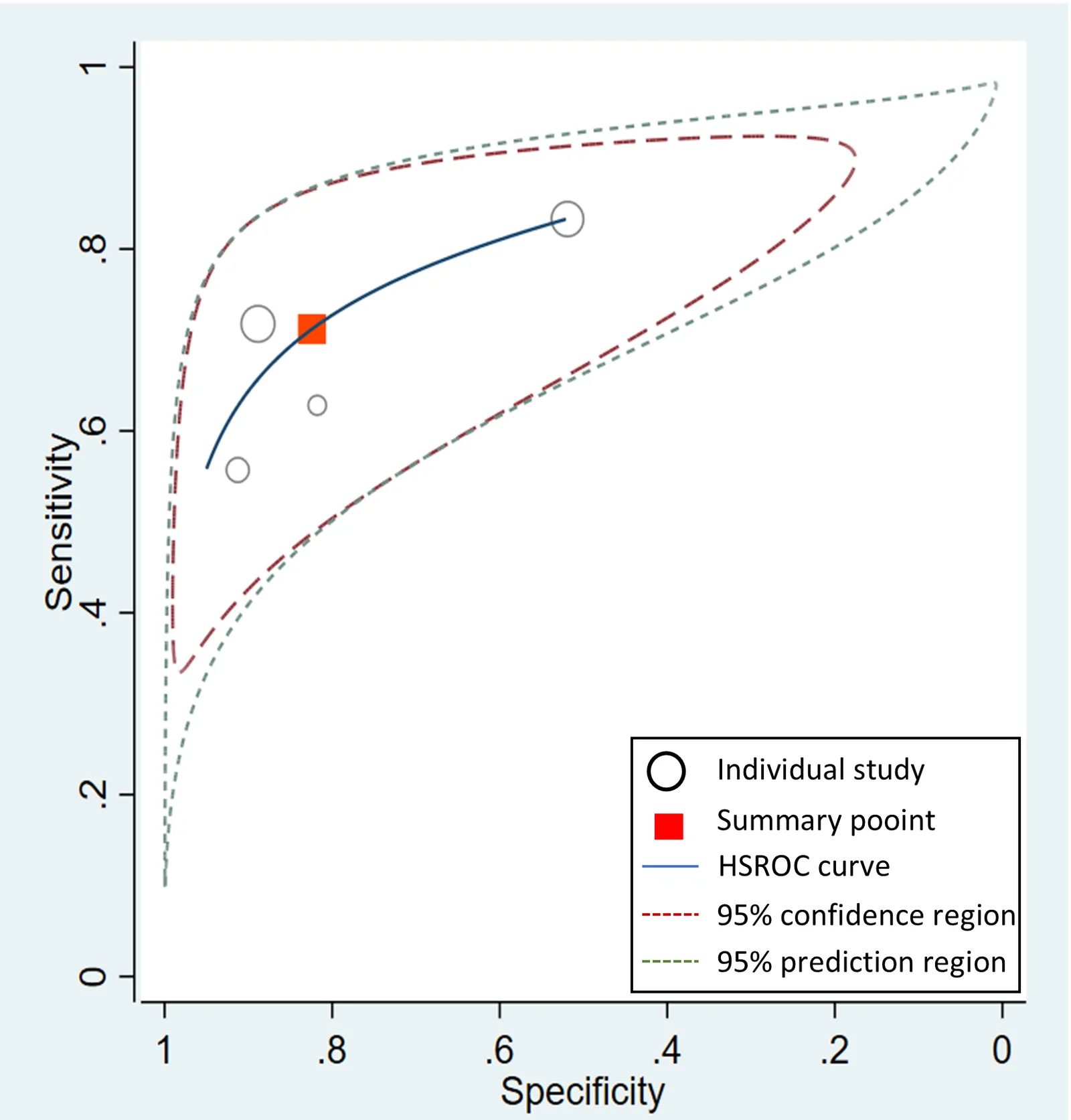
Hierarchical summary receiver-operating characteristic (HSROC) curve for the diagnostic performance of the TyG index for diabetic peripheral neuropathy.

Given that only four studies (43, 45, 46, 48) were included, and substantial heterogeneity was observed across all metrics (sensitivity: *I²* = 78.0%; specificity: *I²* = 95.0%), direct pooling of sensitivity and specificity values was not appropriate. We therefore performed a descriptive synthesis to summarize the diagnostic performance of each study. Yang D et al. (43) compared the diagnostic performance of the TyG index and Neutrophil-to-Lymphocyte Ratio for DPN, and found the TyG index had better predictive ability. At its optimal cut-off value of 9.078, the TyG index achieved a relatively high sensitivity of 0.833 but a low specificity of 0.519, with an AUC of 0.715. Yang NN et al. (48) assessed the TyG index for DPN diagnosis among elderly patients. When the cut-off value exceeded 10.1, the sensitivity decreased to 0.56 while the specificity rose to 0.913, with an AUC of 0.755. Xu F et al. (46) analyzed data from 239 patients with T2DM, and identified 9.85 as the optimal cut-off value, corresponding to a sensitivity of 0.72, specificity of 0.89 and AUC of 0.792. Mai HH et al. (45) compared multiple IR indicators in patients complicated with hypertension and DPN. The TyG index produced the highest AUC of 0.774 at a cut-off value of 9.41, with a sensitivity of 0.629 and specificity of 0.818. We constructed an HSROC curve to evaluate overall discriminative performance (**Figure 4**). The HSROC-AUC was 0.79 (95% CI: 0.73-0.84), indicating that the TyG index exhibited moderate diagnostic accuracy for DPN. Effect points of the four studies clustered near the HSROC curve but were widely scattered. The wide 95% confidence and prediction regions indicated substantial between-study heterogeneity.

## 4 Sensitivity analysis

**Figure 5:**
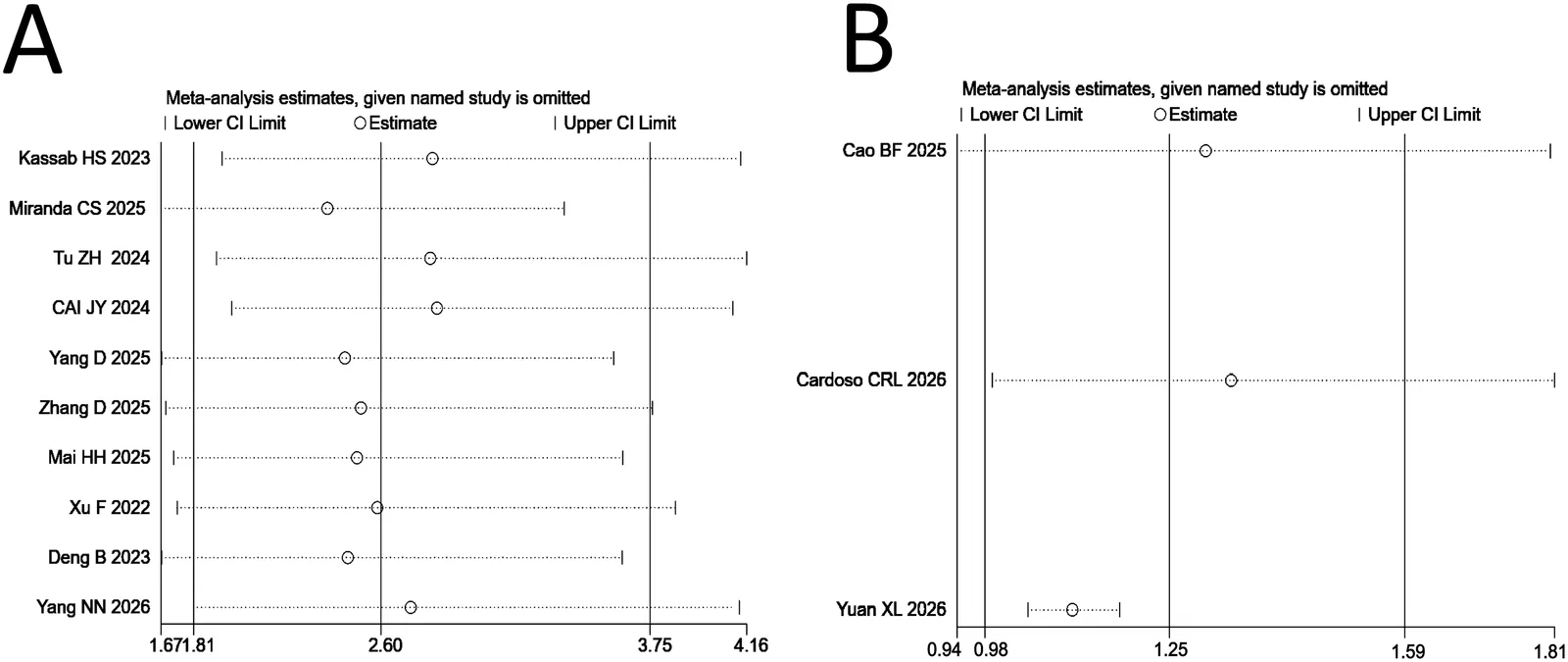
Sensitivity analysis for the association between triglyceride-glucose index and risk of diabetic peripheral neuropathy. **(A)** odds ratio (OR) and **(B)** hazard ratio (HR).

Leave-one-out sensitivity analyses demonstrated that pooled ORs ranged from 2.38 (95% CI: 1.67-3.38) to 2.82 (95% CI: 1.90-4.16) (**Figure 5A**; **Supplementary Table 6**). The direction and statistical significance of the association remained unchanged, indicating robust findings for the OR outcome. Sensitivity analyses of HR outcomes showed that, after excluding either the study of Cardoso CRL et al. (37) or the study of Cao BF et al. (38), the 95% CI of the pooled effect crossed the null value of 1.0, and statistical significance was lost. Conversely, when only the study of Yuan XL et al. (39) was omitted, the pooled estimate remained statistically significant (**Figure 5B**, **Supplementary Table 6**). These findings indicate that the pooled HR estimate is susceptible to the influence of individual studies, reflecting limited robustness.

## 5 Publication bias

**Figure 6:**
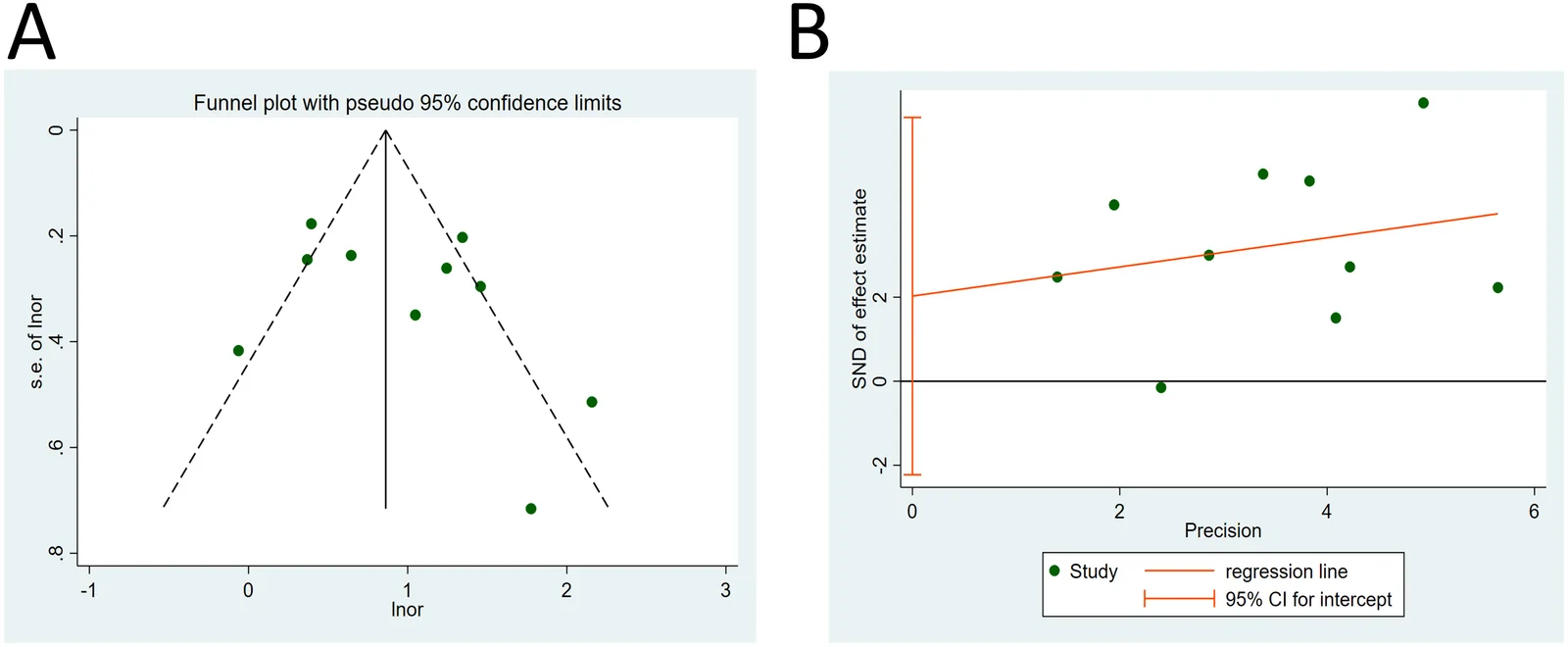
Publication bias for the association between triglyceride-glucose index and diabetic peripheral neuropathy. **(A)** funnel plots of odds ratio (OR) and **(B)** Egger’s test of OR.

Ten cross-sectional studies (22, 23, 41–48) reported OR effect sizes and were eligible for publication bias evaluation. The funnel plot (**Figure 6A**) showed mild asymmetry, suggesting possible publication bias. Egger’s test (t = 1.1, *P* = 0.304; **Figure 6B**) was not statistically significant, confirming the absence of obvious publication bias. Overall, the combination of graphical and quantitative test results indicated that publication bias had a minor impact on the robustness of the study’s conclusions.

## 6 Discussion

### 6.1 Summary of results

This systematic review and meta-analysis synthesized data from 14 observational studies involving 32,281 patients with T2DM. To our knowledge, this is the first study to comprehensively and quantitatively assess the association between the TyG index and both the risk and predictive value of DPN. Our findings revealed that a higher TyG index was associated with an elevated risk of DPN, with pooled estimates of OR 2.614 (95% CI: 1.788-3.821), HR 1.248 (95% CI: 1.003-1.553), and MD 0.42 (95% CI: 0.26-0.58). These results provide evidence-based support for utilizing the TyG index as a simple screening tool for DPN.

IR, which is the core pathological basis for the development and progression of T2DM, can be induced by glucose and lipid metabolic disorders, oxidative stress, and chronic inflammatory infiltration; these factors interact and jointly mediate the pathological progression of various diabetic microvascular complications (49). DPN, a severely disabling microvascular complication, is not exclusively mediated by hyperglycemic toxicity. IR in neural tissue also plays a critical regulatory role by inducing neuronal injury, axonal atrophy, and myelin abnormalities (50). Contemporary evidence indicates that metabolic risk factors, including obesity, dyslipidemia, and hypertension, are not only linked to aggravated IR but also closely associated with a higher risk of DPN (10, 51). Conversely, Pain, sensory disturbances, and impaired balance associated with DPN can lead to muscle wasting, skeletal muscle atrophy, and intermuscular fat infiltration, thereby reducing skeletal muscle glucose uptake and indirectly exacerbating systemic IR (52–54). This interplay establishes a vicious cycle between the two conditions. Collectively, IR serves as a critical intermediary factor driving the onset and progression of DPN and represents an important clinical therapeutic target. A six-year follow-up study by Cho et al. (55) revealed that despite adequate glycemic control, pre-existing IR promoted DPN development and progression. Dyslipidemia was also implicated in disease progression, corroborating that IR influences DPN independently of glycemic status.

The HOMA-IR is a classical, simple measure for assessing IR in epidemiological research. It has been shown to be independently associated with DPN risk (OR = 1.366, 95% CI: 1.093–1.707, *P* = 0.006) (56). Moreover, existing studies have largely included HOMA-IR solely as a confounding variable in analytical models, lacking receiver operating characteristic (ROC) curve analysis to quantify its diagnostic performance, sensitivity, and specificity. Therefore, its practical value for risk stratification in patients with DPN remains unclear (57). In contrast, the present study conducted an exploratory analysis of the diagnostic performance of the TyG index for screening DPN. Four original studies were included, with optimal cut-off values ranging from 9.08 to 10.1 across different populations and AUCs of individual studies ranging from 0.715 to 0.792. We further fitted an HSROC curve yielding an AUC of 0.79 (95% CI: 0.73-0.84), indicating that the TyG index possesses moderate discriminative potential for DPN. Considerable between-study heterogeneity was observed, which may be attributable to multiple factors. On the one hand, the four studies enrolled distinct populations, including general T2DM patients, elderly individuals, and those with hypertension coexisting with DPN. On the other hand, the clinical diagnostic criteria for DPN were not fully standardized across studies (**Supplementary Table 1**). Furthermore, the diagnostic cut-off values of the TyG index differed substantially across studies, showing a trend that sensitivity decreases and specificity rises as cut-off values increase. Due to the limited number of included studies, the statistical power for related tests was insufficient; although no significant threshold effect was detected, its potential influence on the results could not be completely excluded. From a pathophysiological perspective, DPN pathogenesis arises from dual injuries induced by hyperglycemic neurotoxicity and lipotoxicity. Animal studies have demonstrated that lipid metabolic disturbances can induce mitochondrial dysfunction in neuronal axons, disrupt energy metabolism and calcium homeostasis, and impair nerve conduction (58). Meanwhile, hyperglycemia activates the polyol and hexosamine pathways, triggers oxidative stress and inflammatory immune responses, disrupts Schwann cell-axon metabolic coupling, impairs axonal integrity, and drives DPN progression (59). However, HOMA-IR is calculated solely based on fasting glucose and fasting insulin without incorporating lipid-related parameters, and thus may not adequately assess lipid metabolic abnormalities. The TyG index integrates both glycemic and lipid metabolic indicators to comprehensively quantify the severity of glucolipid metabolic disturbances. This characteristic aligns well with the multifactorial pathogenic mechanisms of DPN.

First proposed by Simental-Mendía et al. (25) in 2008, the TyG index has been widely applied to predict the risk of metabolic, renal, cardiovascular, and postoperative complications. For diabetic microvascular complications, numerous studies have identified that a high TyG index is an independent risk factor for diabetic retinopathy and diabetic kidney disease (DKD) (60, 61). Recent clinical data further indicate an association between the TyG index and DPN. Kassab et al. (22) found it to be an independent risk factor for DPN among patients with T2DM, and a cohort study by Yuan et al. (39) drew similar conclusions. Consistent with previous evidence, our meta-analysis also showed that a higher TyG index is associated with increased DPN risk. Subgroup analyses further supported the link between the TyG index and DPN, with a stronger effect observed in Chinese populations. The pooled MD showed no significant difference in non-Asian groups, indicating potential regional and ethnic specificity in the predictive ability of the TyG index. Prior research has reported substantial ethnic differences in IR-related lipid dysregulation. The African natives and individuals of African ancestry included in the non-China cohort presented a typical triglyceride paradox. Despite IR, their triglyceride levels generally remained low or normal. This phenomenon can be explained by higher lipoprotein lipase activity and reduced apolipoprotein CIII, which accelerate the clearance of triglyceride-rich lipoproteins (62). Additionally, compensatory hyperinsulinemia inhibits peripheral lipolysis and free fatty acid release, decreasing substrates for hepatic triglyceride synthesis and preventing excessive hepatic lipid secretion (62). The TyG index is derived from fasting plasma glucose and triglycerides, and its predictive value largely depends on concurrent triglyceride elevation. Its capacity to identify metabolic abnormalities may be limited in individuals with the triglyceride paradox. IR in Chinese populations is frequently accompanied by marked glucolipid metabolic disorders and elevated triglyceride levels (63). Accordingly, the TyG index may effectively reflect the severity of metabolic abnormalities and demonstrate a robust association with DPN. These findings suggest that ethnic metabolic characteristics should be fully considered when applying the TyG index for clinical risk screening. Cut-off values for distinct populations should be established to avoid assessment bias arising from uniform criteria.

Our subgroup analysis showed a greater pooled MD of the TyG index between patients with and without DPN in subjects with BMI < 25 kg/m² versus those with BMI ≥ 25 kg/m², which may be explained by distinct metabolic phenotypes across the BMI spectrum, including metabolically obese normal-weight (MONW), and lean diabetes mellitus. MONW describes individuals with a BMI of 18.5–25 kg/m² and a metabolic syndrome (MetS) score ≥ 3/5 (64, 65). Hermans et al. (65) analyzed 1,244 T2DM patients and found that MONW accounted for 9% of the cohort; the DPN prevalence in this subgroup was 29%, higher than the 18% observed in diabetic patients within the same BMI range without MetS, alongside elevated triglycerides and glycated hemoglobin A1c (HbA1c) and reduced insulin sensitivity. These distinct metabolic derangements may plausibly widen the TyG index MD between patients with and without DPN within this BMI stratum. By contrast, Weyman-Vela et al. (66) categorized individuals with BMI ≥30 kg/m² into two metabolic phenotypes: metabolically healthy obesity (MHO) and metabolically unhealthy obesity (MUO). The TyG index was negatively associated with MHO, whereas the proportion of MUO increased across ascending TyG index quartiles (66). This indicates that baseline TyG levels may be generally elevated among populations with BMI ≥25 kg/m², thereby attenuating the intergroup difference in TyG index between individuals with and without DPN. Lean diabetes mellitus is defined as non-type 1 diabetes occurring in patients with a BMI < 18.5 kg/m² (67). Mohan et al. (67) analyzed 398,258 T2DM patients and found that the DPN prevalence reached 73% in lean diabetics, considerably higher than the rate in patients with higher BMI. Lean patients exhibited lower C-peptide and serum triglyceride levels and a higher proportion of insulin therapy, further enlarging the pooled MD of TyG index between patients with and without DPN within this low-BMI subgroup (67).

The pooled HR from three cohort studies in our meta-analysis showed a positive association between elevated TyG index and the risk of incident DPN; however, sensitivity analysis indicated suboptimal robustness of this finding. Beyond the limited number of included studies and insufficient statistical power, population heterogeneity in pathophysiological profiles may partly account for the instability of these pooled estimates. Zaharia et al. (68) conducted a clustering study reporting baseline DPN prevalence of 36% among newly diagnosed T2DM patients with the severe insulin-deficient diabetes (SIDD) subtype, far exceeding the 17% rate in those with severe insulin-resistant diabetes (SIRD). This suggests that neurotrophic deficiency induced by endogenous insulin shortage may drive DPN progression independent of IR. Although all included cohorts excluded patients with prevalent DPN at baseline, the proportions of participants with SIDD and SIRD subtypes may exhibit interstudy differences. For instance, Garg et al. (69) undertook multi-ethnic cohort studies and found that impaired baseline insulin secretion more strongly predicts T2D in South Asians, while elevated IR constitutes the primary risk factor for diabetes in Black, White, and Pima Indian populations. As a surrogate marker of IR, the TyG index may not effectively capture DPN caused by insulin deficiency. When a cohort contains numerous SIDD patients, the link between TyG index and DPN becomes much weaker, and pooled effect estimates lose stability. Furthermore, some patients experience progressive β**-**cell dysfunction during follow-up, shifting from isolated IR to combined insulin deficiency and further reducing the predictive performance of the TyG index (70). In summary, future cohort studies should measure **β**-cell functional indicators including C-peptide and homeostatic model assessment of β-cell function (HOMA-β) to distinguish the independent contributions of these two pathological pathways, so as to objectively assess the predictive value of the TyG index.

Substantial statistical heterogeneity was observed across the pooled analyses of OR, HR, and MD in this study. Although subgroup and sensitivity analyses preliminarily identified sources of heterogeneity such as geographic region, BMI, and sample size, the heterogeneity remained insufficiently explained due to limitations of the primary data. Drawing on published evidence and clinical characteristics, we further explored potential sources of heterogeneity in an effort to account for the variability in effect sizes across studies. The lack of uniform diagnostic criteria for DPN represents a major source of heterogeneity. To date, there is no universally accepted gold standard for DPN diagnosis, and guidelines from the ADA, IDF, and IWGDF recommend substantially different screening protocols and diagnostic thresholds (71). One comparative empirical study demonstrated that DPN prevalence estimates in the same population could range from 13.1% to 68.5%, depending on the diagnostic guideline adopted (72). Studies enrolled in this meta-analysis adopted diverse diagnostic schemes: nerve conduction velocity tests, clinical symptom and sign scales, and peripheral sensory screening. Some studies relied solely on clinical symptoms and signs for diagnosis, whereas others incorporated objective neurophysiological parameters for quantitative assessment (22, 23, 37, 40, 41, 43–47). These discrepancies in diagnostic accuracy lead to variable DPN detection frequencies. Furthermore, diabetes duration and baseline glycemic control levels may explain part of the observed heterogeneity. The included studies encompassed a wide range of diabetes duration (1.0-15.0 years) (**Table 1**), and diabetes duration has been consistently established as an independent risk factor for DPN (73). Sreedevi et al. (74) conducted a stratified analysis based on T2DM duration, which revealed that DPN prevalence in patients with >15 years of disease reached 61.1%, approximately 1.5 times higher than in those with <5 years, corresponding to a 1.88-fold increased risk of neuropathy. Meanwhile, baseline glycated hemoglobin A1c (HbA1c) and fasting glucose levels, two well-established independent predictors of DPN, varied considerably across the included studies (75, 76). Given that the TyG index is significantly positively correlated with HbA1c, it is plausible that baseline TyG index distributions differ systematically across populations with varying glycemic control statuses. In addition to the aforementioned factors, heterogeneity in baseline medication regimens may also contribute to between-study variability. The included studies did not standardize the use of hypoglycemic or hypolipidemic agents, with participants receiving diverse therapies including metformin, SGLT-2 inhibitors, and statins. A randomized double-blind trial demonstrated that rosuvastatin treatment could improve nerve conduction parameters in DPN patients, and animal experiments have suggested that statins may exert neuroprotective effects by improving microcirculation, reducing oxidative stress products, and inhibiting apoptosis, whereas SGLT-2 inhibitors may confer neuroprotection through modulation of the IGF-1R signaling pathway (77–79). Of note, however, both HbA1c and the use of hypoglycemic or hypolipidemic agents act as metabolic intermediates along the metabolic pathway underlying the association between TyG index and DPN. Previous studies focusing on TyG index and cardiovascular disease have confirmed that HbA1c partially mediates the link between TyG index and adverse outcomes (80). Since hyperglycemia is a major contributor to DPN pathogenesis, HbA1c may play an analogous mediating role in the association between the TyG index and DPN. A prospective NHANES cohort further revealed that antidiabetic and hypolipidemic agents markedly modify the association between the TyG index and mortality: a linear positive correlation was seen in participants not receiving these drugs, while a U-shaped curve was identified in treated individuals, indicating that pharmacotherapy modifies the predictive performance of the TyG index for adverse endpoints (81). Collectively, adjusting for such intermediate variables (HbA1c, hypoglycemic and hypolipidemic drugs) in multivariable models eliminates the association component driven by glucolipid disorders. Causal inference frameworks demonstrate that adjusting for intermediates in the association pathway may generate overadjustment bias and underestimate the overall association between the TyG index and DPN (82). Differences in covariate selection across studies, rather than inadequate adjustment, therefore represent a major source of heterogeneity that biases pooled effect estimates. In the present meta-analysis, most studies adjusted for baseline confounders (age, sex, diabetes duration) to quantify the total association of the TyG index; several further incorporated HbA1c and glucolipid medications without sensitivity analyses to compare different adjustment schemes. This practice exacerbates between-study heterogeneity and impairs the reliability of pooled results.

The aforementioned heterogeneity suggests that the interpretation of the association between the TyG index and DPN warrants caution. Nevertheless, the TyG index retains distinct practical advantages in clinical settings, particularly for longitudinal follow-up and early real-world screening. A unified IR surrogate is critical for objectively evaluating treatment responses during monitoring. The TyG index, which is simple to calculate and cost-effective, lessens the financial cost of repeated measurements and enhances long-term follow-up adherence among patients with chronic diabetes. Serial dynamic monitoring of the TyG index enables clinicians to evaluate metabolic amelioration and individualize antihyperglycemic and hypolipidemic regimens on time. Integrating the TyG index into routine diabetic follow-up can support early DPN risk stratification, addressing the shortage of non-invasive, repeatable screening tools. This strategy may slow early neuromicrovascular injury progression, lower DPN incidence and long-term disability, and is highly translatable for primary clinical practice. However, the TyG index has clear application limitations. As an indirect surrogate of IR, it may not independently diagnose DPN and is not a substitute for standard examinations including nerve conduction studies. Our exploratory diagnostic meta-analysis showed that the optimal TyG index cut-off values reported by original studies for identifying DPN varied widely from 9.08 to 10.10 due to differences in study populations and DPN diagnostic standards. No universal cut-off value for DPN has been validated to date, and large prospective cohorts are needed to establish a standardized cut-off value. Accordingly, the TyG index appears most suitable for population risk screening and serial longitudinal monitoring; clinical decisions should prioritize its dynamic trends instead of single isolated measurements for definitive diagnosis.

### 6.2 Strength and limitation

The limitations of the present study are presented as follows. First, studies included in this meta-analysis were predominantly cross-sectional, with only a small number of cohort studies, leading to limited robustness of the evidence base. Cross-sectional designs fail to clarify the temporal sequence between exposure and outcome and are prone to potential reverse causality bias. Accordingly, the pooled effect sizes derived from the present study merely confirm an association between the TyG index and DPN rather than a causal relationship. Meanwhile, cross-sectional studies cannot determine causal relationships. Consequently, the causal association between the TyG index and DPN could not be fully established, and the impacts of reverse causality and residual confounding factors could not be ruled out. Second, there were variations across individual studies in the cut-off values of the TyG index, diagnostic criteria for DPN, and participant characteristics, which may have introduced clinical heterogeneity. Considerable heterogeneity was observed among the included studies. Although subgroup analyses were performed to explore potential sources of heterogeneity, such as region, sample size, and population characteristics, the small number of studies in certain subgroups led to insufficient statistical power, making it impossible to fully interpret the sources of heterogeneity. Third, most eligible studies focused on Asian populations. Therefore, the applicability of the TyG index in other ethnicities and regions remains to be further verified. Fourth, due to missing data from the original study, we failed to assess the interactive effects of confounding factors, including age, duration of diabetes, gender, and hypoglycemic agents. Fifth, although exploratory analyses of the diagnostic performance of the TyG index identified moderate discriminative ability, these results are constrained by the small number of eligible studies and substantial clinical heterogeneity. Such limitations precluded meaningful meta-regression or subgroup analyses to explore sources of heterogeneity, as well as formal publication bias testing. Accordingly, the pooled diagnostic estimates should be interpreted as preliminary exploratory findings, which merely reflect general trends rather than robust evidence to guide clinical practice. Despite the above limitations, this study possesses several strengths that partially offset its weaknesses. First, our search strategy spanned six major electronic databases and incorporated both Chinese and English literature, enrolling more than 30,000 participants from multicenter, multinational real-world cohorts. The large pooled sample substantially reduced random errors common to small individual studies. Second, this systematic review strictly followed PRISMA reporting guidelines; standardized risk-of-bias tools were used to appraise the methodological quality of included studies, strengthening the reliability of pooled effect sizes. Furthermore, subgroup and sensitivity analyses were implemented to explore potential sources of heterogeneity across multiple dimensions, revealing how population traits, geographic regions and study designs drove discrepancies in effect estimates. While residual confounding cannot be fully ruled out, these rigorous analytical approaches improve the interpretability of our results within the limits of existing data. Finally, this is the first meta-analysis to integrate multi-dimensional quantitative evidence, including ORs, HRs, and MDs, offering tentative insights to address ongoing controversies surrounding the TyG index and DPN. This study systematically revealed the association between the TyG index and the risk of DPN in patients with T2DM. Nevertheless, restricted by the aforementioned limitations, the generalizability of our conclusions is limited. Future large-sample, multicenter prospective cohort studies with unified diagnostic criteria are warranted. Standardized definition of indicators and rigorous adjustment for confounders are required to further clarify the causal relationship between the two indicators. Additionally, stratified analyses covering different ethnicities and metabolic subgroups should be conducted. These efforts will provide more solid and reliable evidence for the application of the TyG index in early risk screening and clinical prevention and management of DPN.

## 7 Conclusion

This meta-analysis revealed that an elevated TyG index was associated with an increased risk of DPN in patients with T2DM. The association presented evident population heterogeneity, which was more pronounced among Chinese populations. Sensitivity analysis confirmed the robustness of the pooled OR, whereas the pooled HR showed limited stability; thus, the causal relationship should be interpreted with caution.

## Data Availability

The original contributions presented in the study are included in the article/**Supplementary Material**. Further inquiries can be directed to the corresponding author.

## References

[1] Burhan A Subpaiboonkit R Huang HC . Global prevalence of diabetes-related neuropathy and vascular complications: a systematic review and meta-analysis. Diabetes Res Clin Pract. (2026) 232:113103. doi: 10.1016/j.diabres.2026.113103 41534596

[2] Pop-Busui R Boulton AJ Feldman EL Bril V Freeman R Malik RA . Diabetic neuropathy: a position statement by the American Diabetes Association. Diabetes Care. (2017) 40:136–54. doi: 10.2337/dc16-2042 27999003 PMC6977405

[3] Carmichael J Fadavi H Ishibashi F Shore AC Tavakoli M . Advances in screening, early diagnosis and accurate staging of diabetic neuropathy. Front Endocrinol (Lausanne). (2021) 12:671257. doi: 10.3389/fendo.2021.671257 34122344 PMC8188984

[4] Hicks CW Wang D Matsushita K Windham BG Selvin E . Peripheral neuropathy and all-cause and cardiovascular mortality in U.S. adults: a prospective cohort study. Ann Intern Med. (2021) 174:167–74. doi: 10.7326/m20-1340 33284680 PMC7932559

[5] Bromberg T Gasquet NC Ricker CN Wu C . Healthcare costs and medical utilization patterns associated with painful and severe painful diabetic peripheral neuropathy. Endocrine. (2024) 86:1014–24. doi: 10.1007/s12020-024-03954-6 39001927 PMC11554691

[6] Zhang Y Fan D Zhang Y Zhang S Wang H Liu Z . Using corneal confocal microscopy to compare Mecobalamin intramuscular injections vs oral tablets in treating diabetic peripheral neuropathy: a RCT. Sci Rep. (2021) 11:14697. doi: 10.1038/s41598-021-94284-4 34282267 PMC8290034

[7] Atmaca A Ketenci A Sahin I Sengun IS Oner RI Erdem Tilki H . Expert opinion on screening, diagnosis and management of diabetic peripheral neuropathy: a multidisciplinary approach. Front Endocrinol (Lausanne). (2024) 15:1380929. doi: 10.3389/fendo.2024.1380929 38952393 PMC11215140

[8] Liaqat A . Effectiveness of current diagnostic methods in the early detection of diabetic peripheral neuropathy: are we missing the window? Cureus. (2026) 18:e106429. doi: 10.7759/cureus.106429 42088795 PMC13138573

[9] Callaghan BC Xia R Reynolds E Banerjee M Burant C Rothberg A . Better diagnostic accuracy of neuropathy in obesity: a new challenge for neurologists. Clin Neurophysiol. (2018) 129:654–62. doi: 10.1016/j.clinph.2018.01.003 29414409 PMC5808853

[10] Elafros MA Andersen H Bennett DL Savelieff MG Viswanathan V Callaghan BC . Towards prevention of diabetic peripheral neuropathy: clinical presentation, pathogenesis, and new treatments. Lancet Neurol. (2022) 21:922–36. doi: 10.1016/s1474-4422(22)00188-0 36115364 PMC10112836

[11] Selvarajah D Kar D Khunti K Davies MJ Scott AR Walker J . Diabetic peripheral neuropathy: advances in diagnosis and strategies for screening and early intervention. Lancet Diabetes Endocrinol. (2019) 7:938–48. doi: 10.1016/s2213-8587(19)30081-6 31624024

[12] Cabre JJ Mur T Costa B Barrio F Lopez-Moya C Sagarra R . Feasibility and effectiveness of electrochemical dermal conductance measurement for the screening of diabetic neuropathy in primary care. Decoding study (Dermal Electrochemical Conductance in Diabetic Neuropathy). J Clin Med. (2019) 8:598. doi: 10.3390/jcm8050598 31052426 PMC6572367

[13] Maalmi H Strom A Petrera A Hauck SM Strassburger K Kuss O . Serum neurofilament light chain: a novel biomarker for early diabetic sensorimotor polyneuropathy. Diabetologia. (2023) 66:579–89. doi: 10.1007/s00125-022-05846-8 36472640 PMC9892145

[14] Määttä LL Andersen ST Parkner T Hviid CVB Bjerg L Kural MA . Serum neurofilament light chain - a potential biomarker for polyneuropathy in type 2 diabetes? Diabetes Res Clin Pract. (2023) 205:110988. doi: 10.1016/j.diabres.2023.110988 38349953

[15] Yang Y Zhao B Wang Y Lan H Liu X Hu Y . Diabetic neuropathy: cutting-edge research and future directions. Signal Transduct Target Ther. (2025) 10:132. doi: 10.1038/s41392-025-02175-1 40274830 PMC12022100

[16] Moriyama K . Mini-review on insulin resistance assessment: advances in surrogate indices and clinical applications. World J Clin cases. (2025) 13:108380. doi: 10.12998/wjcc.v13.i29.108380 40933937 PMC12417990

[17] Samuel VT Shulman GI . The pathogenesis of insulin resistance: integrating signaling pathways and substrate flux. J Clin Invest. (2016) 126:12–22. doi: 10.1172/jci77812 26727229 PMC4701542

[18] Kurz FT Jende JM Salomir R Lovblad KO Lascano A Gariani K . Diabetic peripheral neuropathy: current epidemiology, diagnostic advances, biomarkers, and management strategies. J Diabetes Res. (2026) 2026:e9080699. doi: 10.1155/jdr/9080699 42015589 PMC13100364

[19] Calcutt NA . Diabetic neuropathy and neuropathic pain: a (con)fusion of pathogenic mechanisms? Pain. (2020) 161:S65–s86. doi: 10.1097/j.pain.0000000000001922 32999525 PMC7521457

[20] Guerrero-Romero F Simental-Mendía LE González-Ortiz M Martínez-Abundis E Ramos-Zavala MG Hernández-González SO . The product of triglycerides and glucose, a simple measure of insulin sensitivity. Comparison with the euglycemic-hyperinsulinemic clamp. J Clin Endocrinol Metab. (2010) 95:3347–51. doi: 10.1210/jc.2010-0288 20484475

[21] Cho YK Han KD Kim HS Jung CH Park JY Lee WJ . Triglyceride-glucose index is a useful marker for predicting future cardiovascular disease and mortality in young Korean adults: a nationwide population-based cohort study. J Lipid Atheroscler. (2022) 11:178–86. doi: 10.12997/jla.2022.11.2.178 35656153 PMC9133778

[22] Kassab HS Osman NA Elrahmany SM . Assessment of triglyceride–glucose index and ratio in patients with type 2 diabetes and their relation to microvascular complications. Endocr Res. (2023) 48:94–100. doi: 10.1080/07435800.2023.2245909 37565769

[23] Cai J Peng Y . The clinical significance of PLR, NLR, SII, TyG, and TyG-BMI in peripheral neuropathy among patients with newly diagnosed T2DM. Zhejiang Med J. (2024) 46:2545–8. doi: 10.12056/j.issn.1006-2785.2024.46.23.2023-1297

[24] Page MJ Moher D Bossuyt PM Boutron I Hoffmann TC Mulrow CD . PRISMA 2020 explanation and elaboration: updated guidance and exemplars for reporting systematic reviews. Bmj. (2021) 372:n160. doi: 10.1136/bmj.n160 33781993 PMC8005925

[25] Simental-Mendia LE Rodriguez-Moran M Guerrero-Romero F . The product of fasting glucose and triglycerides as surrogate for identifying insulin resistance in apparently healthy subjects. Metab Syndr Relat Disord. (2008) 6:299–304. doi: 10.1089/met.2008.0034 19067533

[26] Feldman EL Stevens MJ Thomas PK Brown MB Canal N Greene DA . A practical two-step quantitative clinical and electrophysiological assessment for the diagnosis and staging of diabetic neuropathy. Diabetes Care. (1994) 17:1281–9. doi: 10.2337/diacare.17.11.1281 7821168

[27] Bril V Perkins BA . Validation of the Toronto Clinical Scoring System for diabetic polyneuropathy. Diabetes Care. (2002) 25:2048–52. doi: 10.2337/diacare.25.11.2048 12401755

[28] American Diabetes Association Professional Practice Committee . Retinopathy, neuropathy, and foot care: standards of care in diabetes-2025. Diabetes Care. (2025) 48:S252–s65. doi: 10.2337/dc25-S012 39651973 PMC11635040

[29] England JD Gronseth GS Franklin G Miller RG Asbury AK Carter GT . Distal symmetric polyneuropathy: a definition for clinical research: report of the American Academy of Neurology, the American Association of Electrodiagnostic Medicine, and the American Academy of Physical Medicine and Rehabilitation. Neurology. (2005) 64:199–207. doi: 10.1212/01.Wnl.0000149522.32823.Ea 15668414

[30] Truini A Aleksovska K Anderson CC Attal N Baron R Bennett DL . Joint European Academy of Neurology-European Pain Federation-Neuropathic Pain Special Interest Group of the International Association for the Study of Pain guidelines on neuropathic pain assessment. Eur J Neurol. (2023) 30:2177–96. doi: 10.1111/ene.15831 37253688

[31] Association DBoCM . Chinese guidelines for the prevention and treatment of type 2 diabetes (2020 edition) (part 2). Chin J Pract Internal Med. (2021) 41:757–84. doi: 10.19538/j.nk2021090106

[32] Newman L . AHRQ's evidence-based practice centres prove viable. Agency for Healthcare Research and Quality. Lancet. (2000) 356:1990. doi: 10.1016/s0140-6736(05)72963-9 11130533

[33] Stang A . Critical evaluation of the Newcastle-Ottawa scale for the assessment of the quality of nonrandomized studies in meta-analyses. Eur J Epidemiol. (2010) 25:603–5. doi: 10.1007/s10654-010-9491-z 20652370

[34] Khatami R Faghihi M Khatami H Yousefifard M Khatami S . Calculation of sensitivity and specificity from partial data for meta-analyses: introducing some practical methods. Arch Acad Emerg Med. (2025) 13:e56. doi: 10.22037/aaemj.v13i1.2678 40727603 PMC12303406

[35] Wan X Wang W Liu J Tong T . Estimating the sample mean and standard deviation from the sample size, median, range and/or interquartile range. BMC Med Res Methodol. (2014) 14:135. doi: 10.1186/1471-2288-14-135 25524443 PMC4383202

[36] Luo D Wan X Liu J Tong T . Optimally estimating the sample mean from the sample size, median, mid-range, and/or mid-quartile range. Stat Methods Med Res. (2018) 27:1785–805. doi: 10.1177/0962280216669183 27683581

[37] Cardoso CRL Castro GP Leite NC Salles GF . Prognostic value of triglyceride-derived metabolic parameters for micro- and macrovascular complications and mortality in individuals with type 2 diabetes: the Rio de Janeiro type 2 diabetes cohort study. Diabetic Med. (2026) 43:e70263. doi: 10.1111/dme.70263 41714748 PMC12982650

[38] Cao B-F Liu K Chen H-W Xu Z-Y Wang S-A Zhang C-Y . Impact of baseline and trajectory of the cardiometabolic indices on incident microvascular complications in patients with type 2 diabetes. Atherosclerosis. (2025) 407:120407. doi: 10.1016/j.atherosclerosis.2025.120407 40561674

[39] Yuan X Peng M Shi X Yang D Wang F Hou C . Triglyceride-glucose index, genetic susceptibility, and trajectory of microvascular multimorbidity in type 2 diabetes. Sci Rep. (2026) 16:8230. doi: 10.1038/s41598-026-39777-w 41673083 PMC12963423

[40] Sbriscia M Colombaretti D Giuliani A Di Valerio S Scisciola L Rusanova I . Triglyceride glucose index predicts long-term mortality and major adverse cardiovascular events in patients with type 2 diabetes. Cardiovasc Diabetol. (2025) 24:115. doi: 10.1186/s12933-025-02671-2 40065340 PMC11895143

[41] Miranda CS Vj P Miranda C . Correlation of triglyceride-glucose index and the presence of microvascular complications of diabetes. Cureus. (2025) 17:e90363-e. doi: 10.7759/cureus.90363 40978922 PMC12444723

[42] Tu Z Du J Ge X Peng W Shen L Xia L . Triglyceride glucose index for the detection of diabetic kidney disease and diabetic peripheral neuropathy in hospitalized patients with type 2 diabetes. Diabetes Ther. (2024) 15:1799–810. doi: 10.1007/s13300-024-01609-3 38907937 PMC11263315

[43] Yang D Li J Ni Y Zhao J Fang Z . The association of triglyceride-glucose index and neutrophil-to-lymphocyte ratio with type 2 diabetic peripheral neuropathy. Labeled Immunoassays Clin Med. (2025) 32:1228–32.

[44] Zhang D Xi Y . The correlation between TyG index and diabetic peripheral neuropathy. J Jinzhou Med Univ. (2025) 46:51–5. doi: 10.3969/j.issn.2096-305X.2025.03.010

[45] Mai H Li M Zhang M Li H Xue F . The predictive value of different insulin resistance indices and blood pressure variability for peripheral neuropathy in patients with hypertension and diabetes. J Aerospace Med. (2025) 36:662–4.

[46] Xu F Huang X . The predictive value of the triglyceride-glucose index for type 2 diabetes with peripheral neuropathy. J China Prescription Drug. (2022) 20:165–7.

[47] Deng B Hu Y Zhang J Xiao W Li J Hu L . Correlation between triglyceride-glucose index and diabetic vascular complications. Chin J Diabetes. (2023) 31:104–7. doi: 10.3969/j.issn.1006-6187.2023.02.005

[48] Yang N Zheng Z Zhang R Wu Y Wang Q . Correlation of serum UA/HDL-C ratio and TyG index with peripheral neuropathy in elderly patients with type 2 diabetes mellitus and their predictive value. Chin J Gerontol. (2026) 46:2561–4.

[49] Shariat-Madar Z Mahdi F . Potential molecular biomarkers for predicting and monitoring complications in type 2 diabetes mellitus. Molecules. (2025) 30:4448. doi: 10.3390/molecules30224448 41302503 PMC12655058

[50] Grote CW Wright DE . A role for insulin in diabetic neuropathy. Front Neurosci. (2016) 10:581. doi: 10.3389/fnins.2016.00581 28066166 PMC5179551

[51] DeFronzo RA Ferrannini E . Insulin resistance. A multifaceted syndrome responsible for NIDDM, obesity, hypertension, dyslipidemia, and atherosclerotic cardiovascular disease. Diabetes Care. (1991) 14:173–94. doi: 10.2337/diacare.14.3.173 2044434

[52] Purnamasari D Tetrasiwi EN Kartiko GJ Astrella C Husam K Laksmi PW . Sarcopenia and chronic complications of type 2 diabetes mellitus. Rev Diabetes Stud. (2022) 18:157–65. doi: 10.1900/rds.2022.18.157 36309772 PMC9652710

[53] Bittel DC Bittel AJ Tuttle LJ Hastings MK Commean PK Mueller MJ . Adipose tissue content, muscle performance and physical function in obese adults with type 2 diabetes mellitus and peripheral neuropathy. J Diabetes Complications. (2015) 29:250–7. doi: 10.1016/j.jdiacomp.2014.11.003 25547717 PMC4333054

[54] Lopez-Pedrosa JM Camprubi-Robles M Guzman-Rolo G Lopez-Gonzalez A Garcia-Almeida JM Sanz-Paris A . The vicious cycle of type 2 diabetes mellitus and skeletal muscle atrophy: Clinical, biochemical, and nutritional bases. Nutrients. (2024) 16:172. doi: 10.3390/nu16010172 38202001 PMC10780454

[55] Cho YN Lee KO Jeong J Park HJ Kim SM Shin HY . The role of insulin resistance in diabetic neuropathy in Koreans with type 2 diabetes mellitus: a 6-year follow-up study. Yonsei Med J. (2014) 55:700–8. doi: 10.3349/ymj.2014.55.3.700 24719137 PMC3990070

[56] Ma Q . Analysis of factors related to type 2 diabetic peripheral neuropathy based on flash glucose monitoring system. Med Sci Monit. (2023) 29:e939157. doi: 10.12659/msm.939157 37287214 PMC10262751

[57] Matthews DR Hosker JP Rudenski AS Naylor BA Treacher DF Turner RC . Homeostasis model assessment: insulin resistance and beta-cell function from fasting plasma glucose and insulin concentrations in man. Diabetologia. (1985) 28:412–9. doi: 10.1007/bf00280883 3899825

[58] Sajic M Rumora AE Kanhai AA Dentoni G Varatharajah S Casey C . High dietary fat consumption impairs axonal mitochondrial function *in vivo*. J Neurosci. (2021) 41:4321–34. doi: 10.1523/jneurosci.1852-20.2021 33785643 PMC8143198

[59] Feldman EL Nave KA Jensen TS Bennett DLH . New horizons in diabetic neuropathy: Mechanisms, bioenergetics, and pain. Neuron. (2017) 93:1296–313. doi: 10.1016/j.neuron.2017.02.005 28334605 PMC5400015

[60] Yu L Li B . Association between triglyceride-glucose index and diabetic retinopathy: A meta-analysis. Horm Metab Res. (2024) 56:785–94. doi: 10.1055/a-2279-7112 38670124

[61] Deng S Peng L . Triglyceride glucose index and the risk of diabetic nephropathy in patients with type 2 diabetes: A meta-analysis. Horm Metab Res. (2025) 57:106–16. doi: 10.1055/a-2376-6044 39236743

[62] Yu SS Castillo DC Courville AB Sumner AE . The triglyceride paradox in people of African descent. Metab Syndr Relat Disord. (2012) 10:77–82. doi: 10.1089/met.2011.0108 22224930 PMC3311911

[63] Chen Y Zeng H Luo Z Hu H Wang X . Association between the triglyceride-glucose index and risk of type 2 diabetes mellitus and the mediating effect of BMI: a comparative analysis in Chinese and Japanese populations. Front Endocrinol (Lausanne). (2026) 17:1701371. doi: 10.3389/fendo.2026.1701371 41847446 PMC12989386

[64] Pluta W Dudzinska W Lubkowska A . Metabolic obesity in people with normal body weight (MONW)-Review of diagnostic criteria. Int J Environ Res Public Health. (2022) 19:e002261. doi: 10.3390/ijerph19020624 35055447 PMC8776153

[65] Hermans MP Amoussou-Guenou KD Bouenizabila E Sadikot SS Ahn SA Rousseau MF . The normal-weight type 2 diabetes phenotype revisited. Diabetes Metab Syndr. (2016) 10:S82–8. doi: 10.1016/j.dsx.2016.01.035 26960924

[66] Weyman-Vela Y Guerrero-Romero F Simental-Mendía LE . The triglycerides and glucose index is more strongly associated with metabolically healthy obesity phenotype than the lipid and obesity indices. J Endocrinol Invest. (2024) 47:865–71. doi: 10.1007/s40618-023-02201-5 37768526

[67] Mohan V Pramodkumar TA Deepa M Pradeepa R Unnikrishnan R Routray P . Lean diabetes is one end of the spectrum of type 2 diabetes and not a separate entity. Diabetes Res Clin Pract. (2025) 227:112387. doi: 10.1016/j.diabres.2025.112387 40716459

[68] Zaharia OP Strassburger K Strom A Bonhof GJ Karusheva Y Antoniou S . Risk of diabetes-associated diseases in subgroups of patients with recent-onset diabetes: a 5-year follow-up study. Lancet Diabetes Endocrinol. (2019) 7:684–94. doi: 10.1016/s2213-8587(19)30187-1 31345776

[69] Garg R . Insulin resistance versus insulin deficiency: evidence of racial differences in the pathogenesis of type 2 diabetes. BMJ Open Diabetes Res Care. (2021) 9:400. doi: 10.1136/bmjdrc-2021-002261 33771767 PMC8006842

[70] Cui D Feng X Lei S Zhang H Hu W Yang S . Pancreatic β-cell failure, clinical implications, and therapeutic strategies in type 2 diabetes. Chin Med J (Engl). (2024) 137:791–805. doi: 10.1097/cm9.0000000000003034 38479993 PMC10997226

[71] Schaper NC van Netten JJ Apelqvist J Bus SA Hinchliffe RJ Lipsky BA . Practical guidelines on the prevention and management of diabetic foot disease (IWGDF 2019 update). Diabetes Metab Res Rev. (2020) 36:e3266. doi: 10.1002/dmrr.3266 32176447

[72] Chicharro-Luna E Pomares-Gómez FJ Ortega-Ávila AB Coheña-Jiménez M Gijon-Nogueron G . Variability in the clinical diagnosis of diabetic peripheral neuropathy. Prim Care Diabetes. (2020) 14:53–60. doi: 10.1016/j.pcd.2019.05.008 31208891

[73] Lawal Y Mshelia-Reng R Omonua SO Odumodu K Shuaibu R Itanyi UD . Predictors of peripheral neuropathy among persons with diabetes mellitus: a multicenter cross-sectional study. J Am Podiatr Med Assoc. (2025) 115:I22-053. doi: 10.7547/l22-053 40127007

[74] Sreedevi A Pillai GS Sathish S Numpeli M Menon VB Varughese SA . Prevalence and determinants of complications of type 2 diabetes in a community screening program in Kerala. BMJ Public Health. (2025) 3:e002333. doi: 10.1136/bmjph-2024-002333 40521334 PMC12164304

[75] Uslu MF Yılmaz M . Are inflammatory markers important for assessing the severity of diabetic polyneuropathy? Med (Kaunas). (2025) 61. doi: 10.3390/medicina61030400 40142211 PMC11944140

[76] Bas Aksu O Ozkara F Emral R Genc V Corapcioglu D Sahin M . Investigation of the relationship between the triglyceride-glucose index and metabolic and hematological parameters in obese individuals. Turk J Med Sci. (2025) 55:710–8. doi: 10.55730/1300-0144.6019 40686695 PMC12270320

[77] Hernández-Ojeda J Román-Pintos LM Rodríguez-Carrízalez AD Troyo-Sanromán R Cardona-Muñoz EG Alatorre-Carranza MP . Effect of rosuvastatin on diabetic polyneuropathy: a randomized, double-blind, placebo-controlled Phase IIa study. Diabetes Metab Syndr Obes. (2014) 7:401–7. doi: 10.2147/dmso.S65500 25214797 PMC4159311

[78] Daliri M Johnston TP Sahebkar A . Statins and peripheral neuropathy in diabetic and non-diabetic cases: a systematic review. J Pharm Pharmacol. (2023) 75:593–611. doi: 10.1093/jpp/rgac104 36843566

[79] Kaur P Singh T Jena L Gupta T Rana MK Singh S . Dapagliflozin ameliorate type-2 diabetes associated neuropathy via regulation of IGF-1R signaling. J Neuroimmune Pharmacol. (2025) 20:32. doi: 10.1007/s11481-025-10200-x 40178648

[80] Sun Y Huang J Yu X Meng J Qiu G . Association of the triglyceride glucose index and its related indices with coronary artery disease risk: mediation by glycated haemoglobin. Front Endocrinol (Lausanne). (2026) 17:1809007. doi: 10.3389/fendo.2026.1809007 42039131 PMC13106115

[81] Fang C Peng N Cheng J Zhang X Gu W Zhu Z . The association between TyG index and cardiovascular mortality is modified by antidiabetic or lipid-lowering agent: a prospective cohort study. Cardiovasc Diabetol. (2025) 24:65. doi: 10.1186/s12933-025-02620-z 39920712 PMC11806668

[82] Schisterman EF Cole SR Platt RW . Overadjustment bias and unnecessary adjustment in epidemiologic studies. Epidemiology. (2009) 20:488–95. doi: 10.1097/EDE.0b013e3181a819a1 19525685 PMC2744485

