# Supplementary material for: The association between the triglyceride-glucose index and diabetic peripheral neuropathy: a systematic review and meta-analysis
Source: Front Endocrinol (Lausanne). 2026 Sep 4;17:1901899. doi: 10.3389/fendo.2026.1901899 (PMC13585577; doi:10.3389/fendo.2026.1901899)
Supplement: Supplementary file 1 [file SupplementaryFile1.docx]

**Association between triglyceride glucose index and risk of acute kidney injury in critically ill patients: A systematic review and meta-analysis Supplementary Files**

[Supplementary Table 1: PRISMA 2020 Checklist	1](#_Toc30287)

[Supplementary Table 2:Systematic search detail (record number: 2026.7.29)	5](#_Toc31965)

[Supplementary Table 3: Basic characteristics of included studies	8](#_Toc28488)

Supplementary Table 4: Supplementary Table 5: Results of subgroup analysis (MD)	12

Supplementary Table 4: Supplementary Table 5: Results of subgroup analysis (OR)	13

[Supplementary Table 6: Sensitivity analysis (OR/HR)	14](#_Toc5940)

[Supplementary Fig. 1 Subgroup analysis of DPN (MD) ... ...	15](#_Toc11667)

[Supplementary Fig. 2 Subgroup analysis of DPN (OR).............................................................................................................1](#_Toc23807)8

# Supplementary Table 1: PRISMA 2020 Checklist

| **Section and Topic** | **Item #** | **Checklist item** | **Location where item is reported** |
| --- | --- | --- | --- |
| **TITLE** | | |  |
| Title | 1 | Identify the report as a systematic review. |  |
| **ABSTRACT** | | |  |
| Abstract | 2 | See the PRISMA 2020 for Abstracts checklist. |  |
| **INTRODUCTION** | | |  |
| Rationale | 3 | Describe the rationale for the review in the context of existing knowledge. |  |
| Objectives | 4 | Provide an explicit statement of the objective(s) or question(s) the review addresses. |  |
| **METHODS** | | |  |
| Eligibility criteria | 5 | Specify the inclusion and exclusion criteria for the review and how studies were grouped for the syntheses. |  |
| Information sources | 6 | Specify all databases, registers, websites, organisations, reference lists and other sources searched or consulted to identify studies. Specify the date when each source was last searched or consulted. |  |
| Search strategy | 7 | Present the full search strategies for all databases, registers and websites, including any filters and limits used. | Supplementary Table 2 |
| Selection process | 8 | Specify the methods used to decide whether a study met the inclusion criteria of the review, including how many reviewers screened each record and each report retrieved, whether they worked independently, and if applicable, details of automation tools used in the process. |  |
| Data collection process | 9 | Specify the methods used to collect data from reports, including how many reviewers collected data from each report, whether they worked independently, any processes for obtaining or confirming data from study investigators, and if applicable, details of automation tools used in the process. |  |
| Data items | 10a | List and define all outcomes for which data were sought. Specify whether all results that were compatible with each outcome domain in each study were sought (e.g. for all measures, time points, analyses), and if not, the methods used to decide which results to collect. |  |
|  | 10b | List and define all other variables for which data were sought (e.g. participant and intervention characteristics, funding sources). Describe any assumptions made about any missing or unclear information. |  |
| Study risk of bias assessment | 11 | Specify the methods used to assess risk of bias in the included studies, including details of the tool(s) used, how many reviewers assessed each study and whether they worked independently, and if applicable, details of automation tools used in the process. |  |
| Effect measures | 12 | Specify for each outcome the effect measure(s) (e.g. risk ratio, mean difference) used in the synthesis or presentation of results. |  |
| Synthesis methods | 13a | Describe the processes used to decide which studies were eligible for each synthesis (e.g. tabulating the study intervention characteristics and comparing against the planned groups for each synthesis (item #5)). |  |
|  | 13b | Describe any methods used to tabulate or visually display results of individual studies and syntheses. |  |
|  | 13c | Describe any methods used to tabulate or visually display results of individual studies and syntheses. |  |
|  | 13d | Describe any methods used to synthesize results and provide a rationale for the choice(s). If meta-analysis was performed, describe the model(s), method(s) to identify the presence and extent of statistical heterogeneity, and software package(s) used. |  |
|  | 13e | Describe any methods used to explore possible causes of heterogeneity among study results (e.g. subgroup analysis, meta-regression). |  |
|  | 13f | Describe any sensitivity analyses conducted to assess robustness of the synthesized results. |  |
| Reporting bias assessment | 14 | Describe any methods used to assess risk of bias due to missing results in a synthesis (arising from reporting biases). |  |
| Certainty assessment | 15 | Describe any methods used to assess certainty (or confidence) in the body of evidence for an outcome. | - |
| **RESULTS** | | |  |
| Study selection | 16a | Describe the results of the search and selection process, from the number of records identified in the search to the number of studies included in the review, ideally using a flow diagram. | Figure 1 |
|  | 16b | Cite studies that might appear to meet the inclusion criteria, but which were excluded, and explain why they were excluded. | Figure1 |
| Study characteristics | 17 | Cite each included study and present its characteristics. | Table1,Supplementary Table 3 |
| Risk of bias in studies | 18 | Present assessments of risk of bias for each included study. |  |
| Results of individual studies | 19 | For all outcomes, present, for each study: (a) summary statistics for each group (where appropriate) and (b) an effect estimate and its precision (e.g. confidence/credible interval), ideally using structured tables or plots. | Figure 2-4 |
| Results of syntheses | 20a | For each synthesis, briefly summarize the characteristics and risk of bias among contributing studies. | Figure 2-4 |
|  | 20b | Present results of all statistical syntheses conducted. If meta-analysis was done, present for each the summary estimate and its precision (e.g. confidence/credible interval) and measures of statistical heterogeneity. If comparing groups, describe the direction of the effect. |  |
|  | 20c | Present results of all investigations of possible causes of heterogeneity among study results. | Supplementary Figure 1-2  Supplementary Table 4-5 |
|  | 20d | Present results of all sensitivity analyses conducted to assess the robustness of the synthesized results. | Figure 5  Supplementary Table 6 |
| Reporting biases | 21 | Present assessments of risk of bias due to missing results (arising from reporting biases) for each synthesis assessed. | Figure 6 |
| Certainty of evidence | 22 | Present assessments of certainty (or confidence) in the body of evidence for each outcome assessed. | - |
| **DISCUSSION** | | |  |
| Discussion | 23a | Provide a general interpretation of the results in the context of other evidence. |  |
|  | 23b | Discuss any limitations of the evidence included in the review. |  |
|  | 23c | Discuss any limitations of the review processes used. |  |
|  | 23d | Discuss implications of the results for practice, policy, and future research. |  |
| **OTHER INFORMATION** | | |  |
| Registration and protocol | 24a | Provide registration information for the review, including register name and registration number, or state that the review was not registered. |  |
|  | 24b | Indicate where the review protocol can be accessed, or state that a protocol was not prepared. |  |
|  | 24c | Describe and explain any amendments to information provided at registration or in the protocol. |  |
| Support | 25 | Describe sources of financial or non-financial support for the review, and the role of the funders or sponsors in the review. |  |
| Competing interests | 26 | Declare any competing interests of review authors. |  |
| Availability of data, code and other materials | 27 | Report which of the following are publicly available and where they can be found: template data collection forms; data extracted from included studies; data used for all analyses; analytic code; any other materials used in the review. |  |

# Supplementary Table 2:Systematic search detail (record number: 2026.7.29)

| **The search strategy for PubMed** | | |
| --- | --- | --- |
| **Sequence** | **Search terms** | **Hits** |
| #1 | (((((((((triglyceride-glucose index[Title/Abstract]) OR (triglyceride glucose index[Title/Abstract])) OR (triglyceride[Title/Abstract] AND glucose index[Title/Abstract])) OR (TyG index[Title/Abstract])) OR (triacylglycerol glucose index[Title/Abstract])) OR (triacylglycerol[Title/Abstract] AND glucose index[Title/Abstract])) OR (triacylglycerol-glucose index[Title/Abstract]) ) ) OR (triglyceride glucose[Title/Abstract])) OR (TyG[Title/Abstract]) | 5250 |
| #2 | (((((diabetes mellitus[MeSH Terms]) OR (diabetes mellitus[Title/Abstract])) OR (type 2 diabetes[Title/Abstract])) OR (T2DM[Title/Abstract])) OR (type 1 diabetes[Title/Abstract])) OR (T1DM[Title/Abstract]) | 745550 |
| #3 | (((((((((((((((((((((((((Diabetic Neuropathies[MeSH Terms]) OR (Diabetes Complications[MeSH Terms])) OR (Diabetic Neuropathy[Title/Abstract])) OR (Diabetic Amyotrophy[Title/Abstract])) OR (Diabetic Amyotrophies[Title/Abstract])) OR (Diabetic Autonomic Neuropathy[Title/Abstract])) OR (Diabetic Autonomic Neuropathies[Title/Abstract])) OR (Diabetic Neuralgia[Title/Abstract])) OR (Diabetic Neuralgias[Title/Abstract])) OR (Diabetic Neuropathy, Painful[Title/Abstract])) OR (Diabetic Neuropathies, Painful[Title/Abstract])) OR (Diabetic Polyneuropathy[Title/Abstract])) OR (Diabetic Polyneuropathies[Title/Abstract])) OR (Asymmetric Diabetic Proximal Motor Neuropathy[Title/Abstract])) OR (Diabetic Asymmetric Polyneuropathy[Title/Abstract])) OR (Asymmetric Polyneuropathies, Diabetic[Title/Abstract])) OR (Diabetic Mononeuropathy[Title/Abstract])) OR (Diabetic Mononeuropathies[Title/Abstract])) OR (Symmetric Diabetic Proximal Motor Neuropathy[Title/Abstract]) ) OR (diabetic peripheral neuropathy[Title/Abstract])) OR (DPN[Title/Abstract])) OR (cardiovascular autonomic neuropathy[Title/Abstract])) OR (CAN[Title/Abstract])) OR (diabetic nerve damage[Title/Abstract])) OR (distal symmetric polyneuropathy[Title/Abstract])) OR (DSPN[Title/Abstract]) | 173488 |
| #4 | #2 OR #3 | 750174 |
| #5 | #1 AND #4 | 1288 |
| #6 | #5  Filters: Observational Study | 41 |
| **The search strategy for Web of science** | | |
| **Sequence** | **Search terms** | **Hits** |
| #1 | AB=(triglyceride-glucose index OR triglyceride glucose index OR TyG index OR triacylglycerol glucose index OR triacylglycerol-glucose index OR TyG) | 23301 |
| #2 | TS=(Diabetic Neuropathies) | 43911 |
| #3 | AB=(Diabetic Neuropathies OR Diabetes Complications OR Diabetic Amyotrophy OR Diabetic Amyotrophies OR Diabetic Autonomic Neuropathy OR Diabetic Autonomic Neuropathies OR Diabetic Neuralgia OR Diabetic Neuralgias OR Painful Diabetic Neuropathy OR Painful Diabetic Neuropathies OR Diabetic Polyneuropathy OR Diabetic Polyneuropathies OR Asymmetric Diabetic Proximal Motor Neuropathy OR Diabetic Asymmetric Polyneuropathy OR Diabetic Mononeuropathy OR Diabetic Mononeuropathies OR Symmetric Diabetic Proximal Motor Neuropathy OR diabetic peripheral neuropathy OR DPN OR diabetic nerve damage OR distal symmetric polyneuropathy OR DSPN) | 144370 |
| #4 | #2 OR #3 | 161808 |
| #5 | #1 AND #4 | 642 |
| **The search strategy for EMBASE** | | |
| **Sequence** | **Search terms** | **Hits** |
| #1 | 'triglyceride-glucose index':ab,kw,ti OR 'triglyceride glucose index':ab,kw,ti OR 'TyG index':ab,kw,ti OR 'triacylglycerol glucose index':ab,kw,ti OR 'triacylglycerol-glucose index':ab,kw,ti OR 'TyG':ab,kw,ti | 5904 |
| #2 | 'diabetic neuropathy'/exp OR 'diabetic complication'/exp | 257497 |
| #3 | 'Diabetic Neuropathies':ab,kw,ti OR 'Diabetes Complications':ab,kw,ti OR 'Diabetic Amyotrophy':ab,kw,ti OR 'Diabetic Amyotrophies':ab,kw,ti OR 'Diabetic Autonomic Neuropathy':ab,kw,ti OR 'Diabetic Autonomic Neuropathies':ab,kw,ti OR 'Diabetic Neuralgia':ab,kw,ti OR 'Diabetic Neuralgias':ab,kw,ti OR 'Painful Diabetic Neuropathy':ab,kw,ti OR 'Painful Diabetic Neuropathies':ab,kw,ti OR 'Diabetic Polyneuropathy':ab,kw,ti OR 'Diabetic Polyneuropathies':ab,kw,ti OR 'Asymmetric Diabetic Proximal Motor Neuropathy':ab,kw,ti OR 'Diabetic Asymmetric Polyneuropathy':ab,kw,ti OR 'Diabetic Mononeuropathy':ab,kw,ti OR 'Diabetic Mononeuropathies':ab,kw,ti OR 'Symmetric Diabetic Proximal Motor Neuropathy':ab,kw,ti OR 'diabetic peripheral neuropathy':ab,kw,ti OR 'DPN':ab,kw,ti OR 'diabetic nerve damage':ab,kw,ti OR 'distal symmetric polyneuropathy':ab,kw,ti OR 'DSPN':ab,kw,ti | 24694 |
| #4 | #2 OR #3 | 265898 |
| #5 | #1 AND #4 | 210 |
| **The search strategy for CNKI** | | |
| **Sequence** | **Search terms** | **Hits** |
| #1 | TKA%=('甘油三酯葡萄糖指数'+'甘油三酯葡萄糖乘积'+'TyG指数'+'三酰甘油葡萄糖指数') AND (SU%=糖尿病神经病变' OR TKA%=('糖尿病神经痛'+'对称性糖尿病性近端运动神经病'+'非对称性糖尿病性近端运动神经病'+'糖尿病不对称性多神经病'+'糖尿病单一神经病变'+'单纯糖尿病性单神经病'+'糖尿病自主神经病变'+'疼痛性糖尿病性神经病'+'糖尿病肌萎缩')) | 44 |
| **The search strategy for Wan Fang database** | | |
| **Sequence** | **Search terms** | **Hits** |
| #1 | 主题:(甘油三酯葡萄糖指数 OR 甘油三酯葡萄糖乘积 OR TyG指数 OR 三酰甘油葡萄糖指数) and 主题:(糖尿病神经病变 OR 糖尿病周围神经病变 OR 糖尿病自主神经病变 OR 糖尿病神经痛 OR 糖尿病神经病OR 糖尿病肌萎缩) | 23 |
| **The search strategy for CQVIP database** | | |
| **Sequence** | **Search terms** | **Hits** |
| #1 | (((((题名或关键词=甘油三酯葡萄糖指数 OR 题名或关键词=甘油三酯葡萄糖乘积) OR 题名或关键词=TyG指数) OR 题名或关键词=三酰甘油葡萄糖指数) OR (((摘要=甘油三酯葡萄糖指数 OR 摘要=甘油三酯葡萄糖乘积) OR 摘要=TyG指数) OR 摘要=三酰甘油葡萄糖指数)) AND ((((摘要=糖尿病神经病变 OR 摘要=糖尿病周围神经病变) OR 摘要=糖尿病自主神经病变) OR 摘要=糖尿病神经痛) OR 摘要=糖尿病神经病OR 糖尿病肌萎缩)) | 14 |

# Supplementary Table 3: Basic characteristics of included studies

| **Study** | **country** | **Study design** | **Male** | **Age** | **Sample size** | **TyG analysis type** | **Method of**  **diagnosis** | **AHRQ**  **/NOS** | **Follow-up Duration (years)** | **T2D duration（year）** | **Adjusted confounding factors** |
| --- | --- | --- | --- | --- | --- | --- | --- | --- | --- | --- | --- |
| Cardoso CRL  2026 | Brazilian | Cohort study | 258 | 60.2±9.5 | 667 | Continuous | Moderate neuropathic symptoms, regardless of clinical signs, or mild neuropathic symptoms combined with moderate neuropathic signs | 8 | median follow-up of 10.6 years | 8 (3,15) | age,sex,BMI, SBP,HbA1c ,physical  activity,diabetes duration, pre‐existent  macro‐ and complications, use of  antihypertensive, antidiabetic and  lipid-lowering medications |
| Cao BF  2025 | U.S. and Canada | Cohort study | 3057 | 62.4±6.6 | 5000 | Continuous | MNSI score >2.0, or vibratory sensation loss , pressure sensation loss ,ankle jerk reflexloss | 9 | median follow-up  of 3.69 years | NR | age,sex,race,education level,smoking and drinking status, history of CVD, BMI，baseline eGFR, SBP,DBP and diabetes therapy. |
| Yuan XL  2026 | United Kingdom | Cohort study | 11876 | 61.00(55,65) | 19512 | Continuous | WHO ICD-9/ICD-10 codes | 8 | median follow-up  of 12.9 years | 5 | age, sex, ethnicity, TDI, smoking and drinking status,SBP,DBP, LDL-C, HDL-C, diabetes duration, genetic risk score for T2D, dietary quality, sleep duration, physical activity, and medication use. |
| Sbriscia M  2025 | Italian Republic | Cohort study | NR | 40-87 | 465 | Continuous | abnormal EMG findings. | 9 | median follow-up  of 16.8 years | NR | age,sex,smoking, hypertension, BMI, HbA1c, statin therapy, presence of T2D complications, eGFR, total cholesterol, and hs-CRP |
| Kassab HS  2023 | Egypt | Cross-sectional study | 225 | 54.0 ± 8.6 | 500 | Continuous | MNSIe score > 2 or MNSIq score ≥ 7 | 8 | _ | 9.1 ± 6.8 | diabetes duration,HbA1c,UACR |
| Miranda CS  2025 | India | Cross-sectional study | 61.23 | 61.23 ± 11.85 | 125 | Continuous | abnormalities in light touch, pinprick, vibration sense, temperature sensation, or ankle jerk reflex. | 8 | _ | 7.82 ± 5.10 | age,sex,diabetes duration, hypertension status, BMI |
| Tu ZH  2024 | China | Cross-sectional study | 1185 | 60 (48,66) | 1931 | Categorical | DNS score ≥ 1 or  VPT > 25 V | 9 | _ | 5.25 (0.25,12.41) | sex,age,SBP, HbA1c,TC, HDL-C,LDL-C, drinking and smoking history |
| CAI JY  2024 | China | Cross-sectional study | 221 | 47.48 ± 12.99 | 300 | Continuous | diabetes history+clinical symptoms+ neurological signs+EMG | 8 | _ | 1 | age,sex,BMI，HbA1C，HDL-C，LDL-C，FCP，smoking and drinking history，HOMA-IR |
| Yang D  2025 | China | Cross-sectional study | 152 | 54.37 ± 11.82 | 220 | Continuous | diabetes history+clinical symptoms+  neurological signs+EMG | 6 | _ | NR | unadjusted |
| Zhang D  2025 | China | Cross-sectional study | 224 | 59.86 ± 10.71 | 357 | Continuous | abnormal EMG findings. | 7 | _ | 11.97 ± 6.93 | age,sex,diabetes duration,FPG，HbA1c,FCP，TG，TC，LDL-C，HDL-C |
| Mai HH  2025 | China | Cross-sectional study | 32 | 64.71 ± 8.83 | 68 | Continuous | DN4 questionnaire confirmed diabetic symmetricalneuropathic pain of the extremities; MNSI examination revealed physical signs of peripheral neuropathy; DPNCheck indicated abnormal nerve conduction. | 7 | _ | NR | unadjusted |
| Xu F  2022 | China | Cross-sectional study | 145 | 56(50,68) | 239 | Continuous | DPN: diabetes history+clinical symptoms+  neurological signs+EMG | 7 | _ | NR | unadjusted |
| Deng B  2023 | China | Cross-sectional study | 1798 | 56.97 ± 10.76 | 2790 | Categorical | EMG: ≥2 nerves with abnormal conduction velocity | 8 | _ | 8.51±0.96 | age, sex, diabetes duration，WHR，HbA1c，LDL‐C，SUA |
| Yang NN  2026 | China | Cross-  sectional study | 64 | 66.23±4.47 | 107 | Continuous | diabetes history+clinical symptoms+  neurological signs+EMG | 8 | _ | 8.76±2.82 | diabetes duration, FPG, TG, HDL-C,  SUA,UHR |
| AHRQ, Agency for Healthcare Research and Quality; BioLINCC, Biologic Specimen and Data Repository Information Coordinating Center; BMI, Body mass index; CVD, Cardiovascular disease; DBP, Diastolic blood pressure; DNS, Diabetes Neuropathy Symptom Score; DN4, douleur neuropathique en 4 questions; DPN, Diabetic Peripheral Neuropathy; EMG, electromyography; eGFR, Estimated glomerular filtration rate; FCP, Fasting C-peptide; FPG, Fasting plasma glucose; HbA1c, Glycated hemoglobin A1c; HDL-C, High-density lipoprotein cholesterol; HOMA-IR, Homeostasis model assessment of insulin resistance; hs-CRP, High-sensitivity C-reactive protein; INRCA, Istituto Nazionale di Ricovero e Cura per Anziani; IRCCS, Istituto di Ricovero e Cura a Carattere Scientifico; LDL-C, Low-density lipoprotein cholesterol; MNSI, Michigan Neuropathy Screening Instrument; MNSIe, Michigan Neuropathy Screening Instrument Examination; MNSIq, Michigan Neuropathy Screening Instrument Questionnaire; NOS, Newcastle-Ottawa Scale; NR, Not Reported; SBP, Systolic blood pressure; SUA, Serum uric acid; TC, Total cholesterol; TDI, Tissue Doppler imaging; T2DM,Type 2 Diabetes Mellitus;TyG, triglyceride glucose index; UACR, Urinary albumin-to-creatinine ratio; UHR:SUA/HDL-C Ratio; VPT, Vibration Perception Threshold; WHR, Waist-to-hip ratio; WHO, World Health Organization. | | | | | | | | | | | |

**Supplementary Table 4: Results of subgroup analysis (MD)**

| Subgroup | No. of studies | Mean Difference | 95% confidence interval | *P* value | *I^2^* (%) | *P* value for heterogeneity | *P* value Between groups |
| --- | --- | --- | --- | --- | --- | --- | --- |
| **Age (years)** | | | | | | | |
| ≥60 | 4 | 0.48 | 0.09-0.88 | 0.0155 | 91.7 | < 0.0001 | 0.7852 |
| ＜60 | 4 | 0.43 | 0.31-0.55 | 0.0000 | 64 | 0.0394 |  |
| **Region** | | | | | | | |
| Non-Asian | 2 | 0.17 | -0.06-0.41 | 0.1451 | 94.1 | < 0.0001 | 0.0095 |
| Asian | 6 | 0.50 | 0.42-0.59 | 0.0000 | 17.1 | 0.3035 |  |
| **Sample** | | | | | | | |
| ＜500 | 6 | 0.50 | 0.42-0.59 | 0.0000 | 17.1 | 0.3035 | 0.0095 |
| ≥500 | 2 | 0.17 | -0.06-0.41 | 0.1451 | 94.1 | < 0.0001 |  |
| **Ajust** | | | | | | | |
| Yes | 5 | 0.39 | 0.15-0.62 | 0.0014 | 94.8 | < 0.0001 | 0.4675 |
| No | 3 | 0.49 | 0.36-0.62 | 0.0000 | 0 | 0.4030 |  |
| **BMI(kg/m^2^)** | | | | | | | |
| ＜25 | 2 | 0.79 | 0.38-1.21 | 0.0002 | 30.7 | 0.2296 | 0.0302 |
| ≥25 | 4 | 0.29 | 0.10-0.48 | 0.0026 | 91.9 | < 0.0001 |  |

**Supplementary Table 5:Results of subgroup analysis (OR****)**

| Subgroup | No. of studies | Odds ratio | 95% confidence interval | *P* value | *I^2^* (%) | *P* value for heterogeneity | *P* value Between groups |
| --- | --- | --- | --- | --- | --- | --- | --- |
| **Age（years）** | | | | | | | |
| ≥60 | 4 | 3.002 | 1.289-6.990 | 0.0108 | 75.3 | 0.0069 | 0.9421 |
| ＜60 | 4 | 2.887 | 1.578-5.281 | 0.0006 | 71.4 | 0.0149 |  |
| **BMI（kg/m²）** | | | | | | | |
| ≥25 | 4 | 2.434 | 1.174-5.047 | 0.0168 | 77 | 0.0045 | 0.9563 |
| ＜25 | 4 | 2.366 | 1.177-4.757 | 0.0156 | 76.3 | 0.0054 |  |
| **Sample** | | | | | | | |
| ＜500 | 7 | 3.032 | 1.860-4.944 | 0.0000 | 67 | 0.0058 | 0.3216 |
| ≥500 | 3 | 2.025 | 1.077-3.807 | 0.0284 | 86.6 | 0.0006 |  |
| **Ajust** | | | | | | | |
| Yes | 7 | 2.296 | 1.427-3.694 | 0.0006 | 80 | < 0.0001 | 0.1231 |
| No | 3 | 3.786 | 2.483-5.773 | < 0.0001 | 0 | 0.5425 |  |
| **Region** |  |  |  |  |  |  |  |
| Non-China | 2 | 3.354 | 0.599-18.783 | 0.1686 | 90.5 | 0.0012 | 0.7668 |
| China | 8 | 2.569 | 1.763-3.743 | 0.0000 | 69 | 0.0020 |  |

# Supplementary Table 6: Sensitivity analysis (OR/HR)

| Outcome | Omitted Study | Data with study removed OR/HR (95% CI) | *I^2^* | *P* |
| --- | --- | --- | --- | --- |
| DPN | Kassab HS 2023 | 2.82 [1.93, 4.14] | 71.2 | ＜0.001 |
|  | Miranda CS 2025 | 2.38 [1.67, 3.38] | 73.7 | ＜0.001 |
|  | Tu ZH 2024 | 2.82 [1.90, 4.16] | 75.2 | ＜0.001 |
|  | CAI JY 2024 | 2.84 [1.97, 4.10] | 74.8 | ＜0.001 |
|  | Yang D 2025 | 2.45 [1.67, 3.60] | 75.4 | ＜0.001 |
|  | Zhang D 2025 | 2.52 [1.69, 3.76] | 76.8 | ＜0.001 |
|  | Mai HH 2025 | 2.50 [1.72, 3.63] | 77.3 | ＜0.001 |
|  | Xu F 2022 | 2.59 [1.74, 3.86] | 78.1 | ＜0.001 |
|  | Deng B 2023 | 2.46 [1.67, 3.63] | 73.4 | ＜0.001 |
|  | Yang NN 2026 | 2.73 [1.81, 4.13] | 77.7 | ＜0.001 |
| DPN(HR) | Cardoso CRL 2026 | 1.30[0.94,1.80] | 96.5 | ＜0.001 |
|  | Cao BF 2025 | 1.34[0.99,1.81] | 85.5 | 0.009 |
|  | Yuan XL 2026 | 1.11[1.04,1.17] | 0 | 0.826 |

Supplementary Fig. 1 Subgroup analysis of DPN (MD)

(A) Age; (B) Region ; (C) Sample; (D) Ajust；(E)BMI

Supplementary Fig. 2 Subgroup analysis of DPN (OR)

(A) Age; (B) BMI; (C) Sample; (D) adjust;(E)region
